# Molecular Dynamics Investigation of the Influenza Hemagglutinin Conformational Changes in Acidic pH

**DOI:** 10.1021/acs.jpcb.4c04607

**Published:** 2024-11-04

**Authors:** Shadi A. Badiee, Vivek Govind Kumar, Mahmoud Moradi

**Affiliations:** Department of Chemistry and Biochemistry, University of Arkansas, Fayetteville, Arkansas 72701, United States

## Abstract

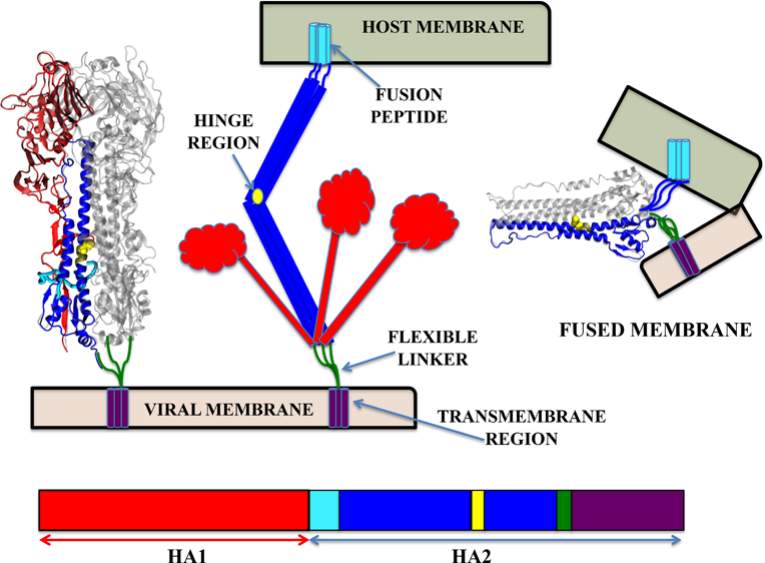

The surface protein hemagglutinin (HA) of the influenza virus plays a pivotal role in facilitating viral infection by binding to sialic acid receptors on host cells. Its conformational state is pH-sensitive, impacting its receptor-binding ability and evasion of the host immune response. In this study, we conducted extensive equilibrium microsecond-level all-atom molecular dynamics (MD) simulations of the HA protein to explore the influence of low pH on its conformational dynamics. Specifically, we investigated the impact of protonation on conserved histidine residues (H106_2_) located in the hinge region of HA2. Our analysis encompassed comparisons between nonprotonated (NP), partially protonated (1P, 2P), and fully protonated (3P) conditions. Our findings reveal substantial pH-dependent conformational alterations in the HA protein, affecting its receptor-binding capability and immune evasion potential. Notably, the nonprotonated form exhibits greater stability compared to protonated states. Conformational shifts in the central helices of HA2 involve outward movement, counterclockwise rotation of protonated helices, and fusion peptide release in protonated systems. Disruption of hydrogen bonds between the fusion peptide and central helices of HA2 drives this release. Moreover, HA1 separation is more likely in the fully protonated system (3P) compared to nonprotonated systems (NP), underscoring the influence of protonation. These insights shed light on influenza virus infection mechanisms and may inform the development of novel antiviral drugs targeting HA protein and pH-responsive drug delivery systems for influenza.

## Introduction

Thousands of people suffer from influenza infections every year, but there is not any fundamental cure for them. Therefore, it is necessary to thoroughly understand the infection process and the critical keys to preventing infections. An influenza virus can be classified into four subtypes: A, B, C, and D. Those belonging to groups A and B contain two membrane-embedded glycoproteins named hemagglutinin (HA) and neuraminidase (NA), while those belonging to groups C and D contain only one surface glycoprotein, known as hemagglutinin-esterase fusion (HEF).^1−5^ Influenza A and B viruses are responsible for most human infections and are responsible for seasonal epidemics and occasional pandemics. The influenza virus uses glycoproteins on its surface to enter host cells,^3^ and understanding the structural features of these proteins that make them vulnerable to inhibitors is important for the development of antiviral drugs.^6,7^ As part of the fusion process, viral hemagglutinin (HA) plays an important role.^8^ Influenza viruses have been very widely studied and are well characterized as a global health concern. However, the atomistic details of influenza HA-mediated membrane fusion are still poorly understood.^9,10^

**Figure 1:**
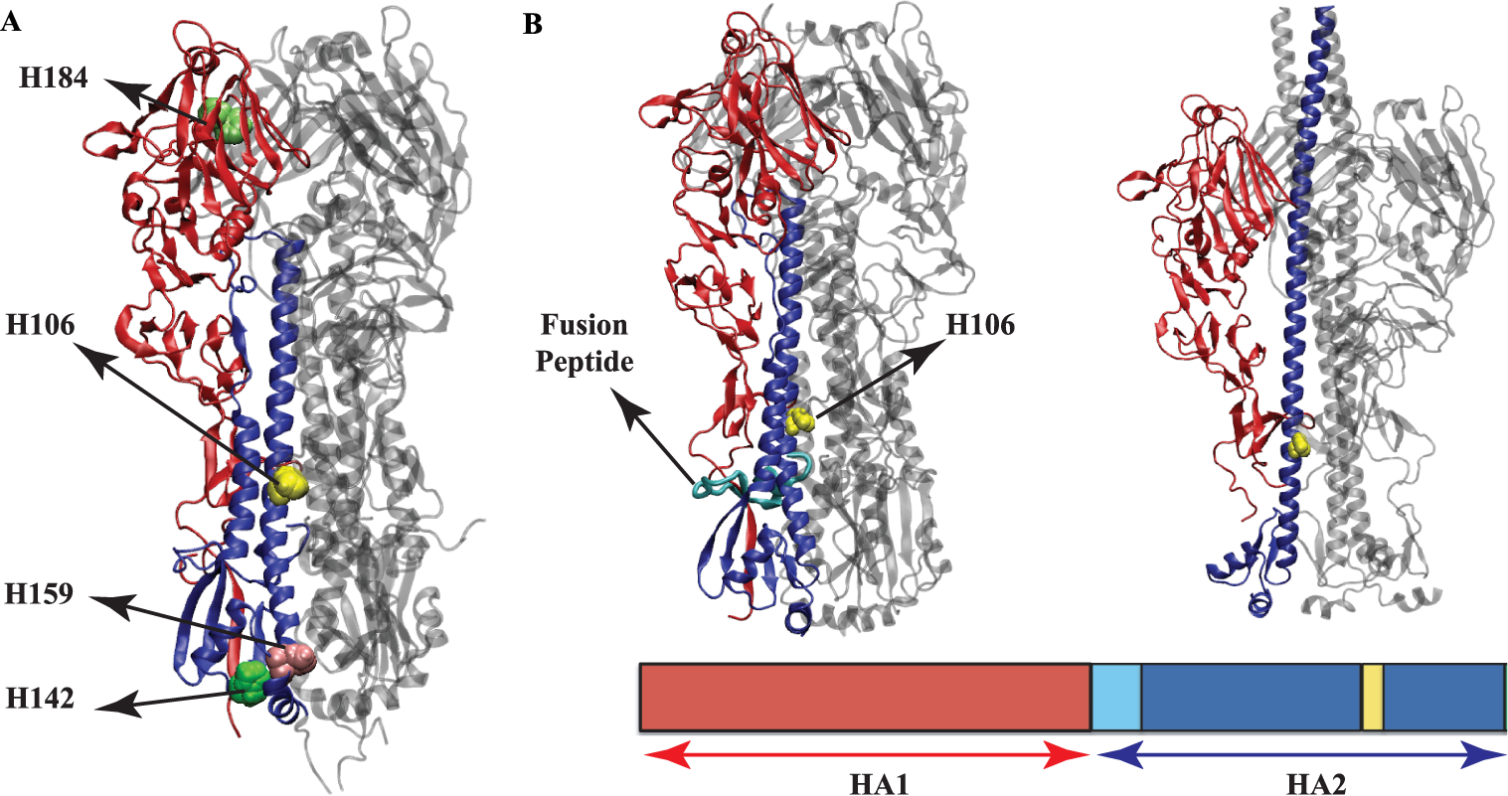
(A) HA structure showing histidine residues (H106_2_, H142_2_, H159_2_ and H184_1_) thought to play a key role in pH-induced conformational changes. (B) A representation of influenza HA protein-mediated membrane fusion: At low pH, the HA1 domains dissociate from the HA2 domains, exposing the fusion peptide. Histidine 106_2_ (H106_2_), shown in yellow, is located in the hinge region of HA2.

Hemagglutinin (HA) plays a key role in the virus’s ability to infect host cells.^11−14^ Hemagglutinin binds to sialic acid receptors on the surface of viral membrane, which allows the virus to enter and replicate within host cells.^15^ Hemagglutinin (HA) protein is synthesized as a precursor protein, known as HA0, which is then cleaved by a cellular protease to generate the mature HA protein.^13,16,17^ The hemagglutinin (HA) has a trimeric structure composed of three identical monomers, each monomer is composed of two main subunits, HA1 and HA2 (Figure 1). These two subunits are covalently held together by disulfide bond^18^ and form the functional unit of the protein. HA1 forms the globular head of the protein which is responsible for binding to host cell receptors and initiating the viral entry into the host cell.^13,19^ In comparison to HA1, HA2 is smaller. It contains the fusion machinery and is responsible for anchoring the HA protein to the viral envelope. It is composed of the stem domain which is important for the stability of the protein and the interactions between the HA and the other viral proteins.^13,20,21^ The HA2 subunit comprises the fusion peptide (FP, residues 1–20), two β-strands (TBS, residues 21–37), helix A (residues 38–54), B loop (residues 55–75), coiled coil (residues 76–104), hinge region (residues 105–129), and ectodomain and transmembrane domain (TMD, residues 130–175).^22,23^ Additionally, domains in HA1 include the fusion (F′), vestigial esterase (VE), and receptor-binding domain (RBD).^24,25^ The RBD contains the receptor-binding site (RBS), which is made up of four secondary structural elements: the 130-loop, the 150-loop, the 190-helix, and the 220-loop.^26^ The VE domain encompasses the 30-loop, which consists of HA1 residues 22–37.^27^ Throughout this paper, subscript 1 will be used for residues belonging to HA1, while subscript 2 will denote residues of HA2.

In response to acidic conditions or low pH environment, hemagglutinin (HA), undergoes extensive conformational changes that allow the N-terminal portion of the HA2 subunit, called FP, to be inserted into the membrane of the host endosome.^21,28−32^ HA1 is thought to move away from HA2, causing the exposure of a FP at the HA2 N-terminus.^16,17,27^ HA2 is then thought to bend at a hinge region, which eventually results in the lipid bilayers being pulled toward each other.^16,17^ The HA2 C-terminus consists of a transmembrane domain needs to span the bilayer in order to promote fusion.^33^ Alternatively, upon acidification, the major conformational change happen in HA2. The B-loop located between two helices in HA2 has a loop-to-helix transformation and forms an α-helix elongating the fusion peptide to the endosomal membrane^34−37^ (Figure 1). Experimental investigations have utilized point mutations within both complete hemagglutinin (HA) and isolated fusion peptides in lipid mixing assays to explore their effects.^28,37−42^ Recent experimental evidence has shown that multiple HA trimers work cooperatively to cause membrane fusion.^43^ Studies have shown that the ideal pH for HA conformational changes is between 4.8 and 6.0.^18,44^ Histidine depends on degree of conservation and its position on HA protein was identified as a potential pH-sensing residue.^45^ Histidine is the only amino acid with a p*K*_a_ value similar to the acidic pH required for HA-mediated membrane fusion. Therefore, conformational changes of proteins that require acidic environments can be triggered by protonating one or more histidines in their structures.^45−47^ Therefore, H184_1_ on HA1 and H106_2_, H111_2_, H142_2_, and H159_2_ on HA2 were suggested as conserved histidines that can potentially trigger the conformational change mediating membrane fusion in HA protein^45^ (Figure 1A). HA’s fusion domain consists of all the proposed histidines except for H184_1_.^48^ Among these histidine residues, a detailed analysis has been conducted for H111_2_ and H106_2_ in subtype H3 and H5, respectively.^48−51^ Studies indicated that conformational changes on HA is increased through protonation of H106_2_/H111_2_. Additionally, several studies have demonstrated that mutations in residues located near the buried fusion peptide can affect the stability of HA.^12,50,52−55^ For instance, in H3 hemagglutinin, H106_2_ forms a hydrogen bond with K51_2_. Substituting histidine with phenylalanine at this position has been shown to slightly lower the fusion pH, indicating that the mutation disrupts key interactions required for conformational changes during the fusion process.^48,50^ Furthermore, research on H3 and H7 hemagglutinins has indicated that protonating the H106_2_ residue within the fusion pH range is likely to compromise structural integrity, potentially facilitating the release of the fusion peptide and triggering the subsequent process by initiating B-loop refolding.^56^

Similarly, an experimental study by Trost et al. (2019)^57^ explored the role of a conserved histidine at position 111 of HA2 in Group-1 influenza hemagglutinin (HA), which is crucial for membrane fusion. In their experiments, the authors substituted H111_2_ with alanine (H111A) and observed a complete loss of fusion activity. Since alanine cannot be protonated, the mutation disrupted the pH-dependent conformational changes necessary for fusion. These findings demonstrate that H111_2_ plays an essential role in initiating acid-induced structural changes, much like H106_2_ in H3 hemagglutinin, and suggest that it could serve as a potential target for antiviral therapies aimed at inhibiting the fusion process.

A number of virus groups with pH-dependent infectivity have provided evidence to support the histidine-switch hypothesis, which states that changes in pH can alter the conformational state of the virus proteins, leading to altered infectivity.^45,47,58−67^

In this study, we investigated the conformational rearrangements of influenza HA when it is exposed to an acidic environment, with a specific focus on the protonation of conserved HA2 hinge histidine residues (H106_2_). Histidine is an amino acid with a p*K*_a_ close to the acidic pH required for HA-mediated membrane fusion, making it a critical residue in triggering conformational changes. Notably, the histidine residue H106_2_ is located in the hinge region, a pH-sensitive region crucial in the fusion process. We assessed the protonation potential of various histidine residues located on HA2 (H106_2_, H142_2_, H159_2_) using Propka,^69^ which revealed that H106_2_ had the highest probability of its p*K*_a_ going above 7.4, despite fluctuations in p*K*_a_ values. As a result, H106_2_ became the primary focus of our study. While pH changes can affect multiple residues, we specifically examined the effects of protonating one, two, or all three histidines (H106_2_). All-atom equilibrium MD simulations at the microsecond level were employed to study these effects. Our simulations, while extensive, are limited by their duration, which constrains the extent of conformational changes that can be observed. Nevertheless, the data reveal that the HA protein undergoes conformational changes even when a single histidine residue (H106_2_) is protonated. We acknowledge the limitations of simulation time and methodology but believe our findings provide valuable insights into the role of histidine protonation in driving conformational changes in HA.

## Methods

Our simulations were conducted based on the crystal structure of the Group-2 HA (PDB entry: 5KUY).^68^ We first used Propka^69^ to determine whether any of the titratable residues could potentially have a nondefault protonation state. This is the protocol we typically use for MD and the outcome was that all titratable residues have their default protonation state. The initial analysis indicated that all titratable residues maintained their default protonation state, and therefore, no histidines were protonated prior to the simulations. Under this condition, we performed three independent repeats of simulations, referred to as the NP simulations. Subsequently, Propka^69^ was used again on the resulting trajectories, with a specific focus on the three conserved histidine residues (H106_2_, H149_2_, and H159_2_). Throughout the simulations, the p*K*_a_ values of these residues varied but generally remained below 7.4. However, there were instances where the p*K*_a_ exceeded 7.4. We calculated the probability of the p*K*_a_ value going above 7.4 for the three aforementioned histidines in all three repeats and all three protomers. The only histidine with a significant probability of its p*K*_a_ exceeding 7.4 in at least one protomer and one repeat is H106_2_, where the probability reaches nearly 40% (Figure S1). In contrast, for the other histidines, the highest observed probability is only around 5%. Based on these findings, alongside existing literature, we focused on the protonation state of conserved histidine residue (H106_2_ which is H435 in PDB file), located in the hinge region, while acknowledging that other histidines may also contribute to the hemagglutinin (HA) fusion process. In the Results section, HA2 residues were renumbered for the sake of convenience and consistency with previous studies, despite having different numbers in the PDB file. For instance, residue H435 in the PDB file was renumbered as residue H106_2_ in this study to align with established literature conventions. Throughout this paper, subscript 1 will be used for residues belonging to HA1, while subscript 2 will denote residues of HA2. To generate simulation inputs, we utilized the CHARMM-GUI simulation input generator.^70,71^ To simulate the effects of a low pH environment, the hinge histidine (H106_2_) was configured to have two protons and be positively charged. In our simulations, we used two distinct protonation states to represent neutral and acidic conditions. At neutral pH, the proton is located on the N_δ_ atom of the imidazole ring, corresponding to the HSD state. For simulations mimicking low pH, an additional proton is placed on the N_ϵ_ atom, representing the HSP state. Each peptide model was therefore constructed with histidines in either the HSD state (neutral pH) or the HSP state (acidic pH), which accurately reflects the different environmental conditions. Four different systems were constructed to examine the effects of histidine protonation on the protein. The no protonation (NP) system features three H106_2_ residues with each residue in the HSD state, having one proton on the N_δ_ atom of the imidazole ring. The single protonation (1P) system has one of the three H106_2_ residues doubly protonated (HSP state), with two protons on the imidazole ring, while the remaining two residues are in the HSD state. The two protonations (2P) system includes two out of the three H106_2_ residues in the HSP state, with each of these residues having two protons, while the third residue remains in the HSD state. Lastly, the three protonations (3P) system features all three H106_2_ residues doubly protonated in the HSP state, with two protons on the imidazole ring. For each system, three replicate simulations were conducted to ensure reliable results.

Each system comprised one protein, 0.15 M NaCl, and TIP3P water molecules.^72^ For systems with no protonation (NP) and one protonation (1P), each contained 138 281 water molecules and had dimensions of 163 × 163 × 163 Å^3^. Systems with two protonations (2P) and three protonations (3P) included 130 646 water molecules and had dimensions of 160 × 160 × 160 Å^3^. To describe all molecules accurately, we employed the CHARMM36m all-atom additive force field parameters.^73,74^ Preliminary MD simulations were conducted using NAMD 2.13^75^ before the production runs on Anton 2. During simulations, we employed a Langevin integrator with periodic boundary conditions at a temperature of 310 K, a collision frequency of 0.5/ps, and a time step of 2 fs. The pressure was maintained at 1 atm using the Nosé–Hoover Langevin piston method.^76,77^ For nonbonded interactions, a smoothed cutoff distance of 10–12 Å was applied, and long-range electrostatic interactions were calculated using the particle mesh Ewald (PME) method.^78^ Prior to equilibration, each system underwent energy minimization for 10,000 steps using the conjugate gradient algorithm.^79^ Equilibration consisted of an initial relaxation in the NVT ensemble followed by a 15 ns equilibrium simulation in the NPT ensemble. After equilibration, production runs were performed on Anton 2 for 2.4 μs with a time step of 2.4 fs. Two additional repetitions were carried out for all systems (NP, 1P, 2P, and 3P) on Anton2, referred to as repeat 2 and repeat 3 in the manuscript. To generate initial conformations for repeat 2, the original production run for each model was extended by 0.5 ns on TACC Stampede. Subsequently, the production runs were extended by an additional 0.5 ns to generate the initial conformations for repeat 3. In total, 28.8 μs of simulation data was generated across 12 systems, with 2.4 μs allocated to each of the systems. For the simulations on Anton2, pressure was maintained isotropically at 1 atm using the MTK barostat, and temperature was controlled at 310 K using the Nosé–Hoover thermostat.^76,77^ Long-range electrostatic interactions were computed using the FFT (fast Fourier transform) method implemented on Anton 2.^80^

For analysis, molecular snapshots, and visualization, we employed visual molecular dynamics (VMD).^81^ Hydrogen bonds and salt bridges were calculated using VMD plugins with cutoff distances of 4.0 Å and angles of 30° for hydrogen bonds. Salt bridge distances were computed as the smallest distance between donor and acceptor atoms. Principal component analysis (PCA) was performed using PRODY software,^82^ considering only Cα. To compare the major modes of variation between protonated and nonprotonated conditions, principal component analysis (PCA) was performed separately for each condition. First, the trajectories for the protonated and nonprotonated systems were separated, specifically focusing on the fusion peptide (FP) residues. We then combined the trajectory files for all nonprotonated segments and performed a similar step for all protonated segments. Each set of trajectories was aligned based on the FP residues to ensure consistent structural orientation. PCA was then applied to the aligned trajectories to identify the dominant modes of motion. The results were projected onto the principal component (PC1, PC2) space. Separate PCA plots were generated for the NP system (nonprotonated), 3P system (fully protonated), and for systems with partial protonation (1P, 2P), which show a combination of protonated and nonprotonated segments, represented in two sections.

The rotation and tilt angles of specific protein segments were calculated using the direction of the roll axis (third principal axis). For the rotation angle, for each frame in the trajectory, the protein was aligned to the first frame to ensure consistency in angle measurements. The roll axis of the selected segment was identified at frame 0 and at any given frame. The rotation angle was defined as the angle between the roll axes at frame 0 and at any given frame. Additionally, the tilt angle were calculated by measuring the angle between the roll axis of the selected segment and the HA2 protein.

## Results and Discussion

**Figure 2:**
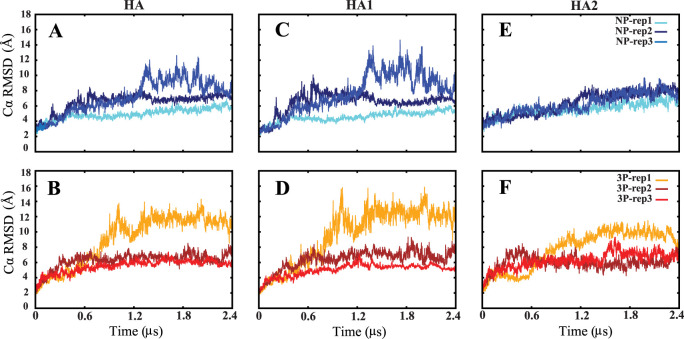
Cα RMSD time series of nonprotonated (NP) and fully protonated (3P) systems of HA (A, B), HA1 (C, D), and HA2 (E, F). Each NP and 3P system simulation was run three times for 2.4 μs each.

The RMSD plots (Figure 2) present the RMSD values for HA and its individual domains, HA1 and HA2, throughout the simulations. The complexity and size of the HA protein pose a significant obstacle in obtaining convergence in our simulations, which last for a few microseconds. This indicates that additional simulation time is necessary to accurately capture the protein’s complete conformational changes and ensure reproducibility of the results. Despite the limited simulation time, we can still observe significant conformational changes and deviations in both the NP and 3P systems. Notably, the 3P systems exhibit higher conformational deviations compared to the NP systems. This increased deviation in the 3P systems suggests that the protonation state significantly influences the dynamic behavior of the protein. To achieve true convergence and reproducibility, longer simulation times are required. However, within the constraints of the current simulation time, we have focused our analysis on specific protein regions. By doing so, we can provide a detailed examination of the conformational changes occurring in these regions. This targeted approach allows us to draw meaningful conclusions about the behavior of these key regions, even if a comprehensive understanding of the entire protein’s dynamics is not yet attainable.

### Conformational Dynamics of the S4 Helix in Fully Protonated (3P) Compared to Nonprotonated (NP) Systems

**Figure 3:**
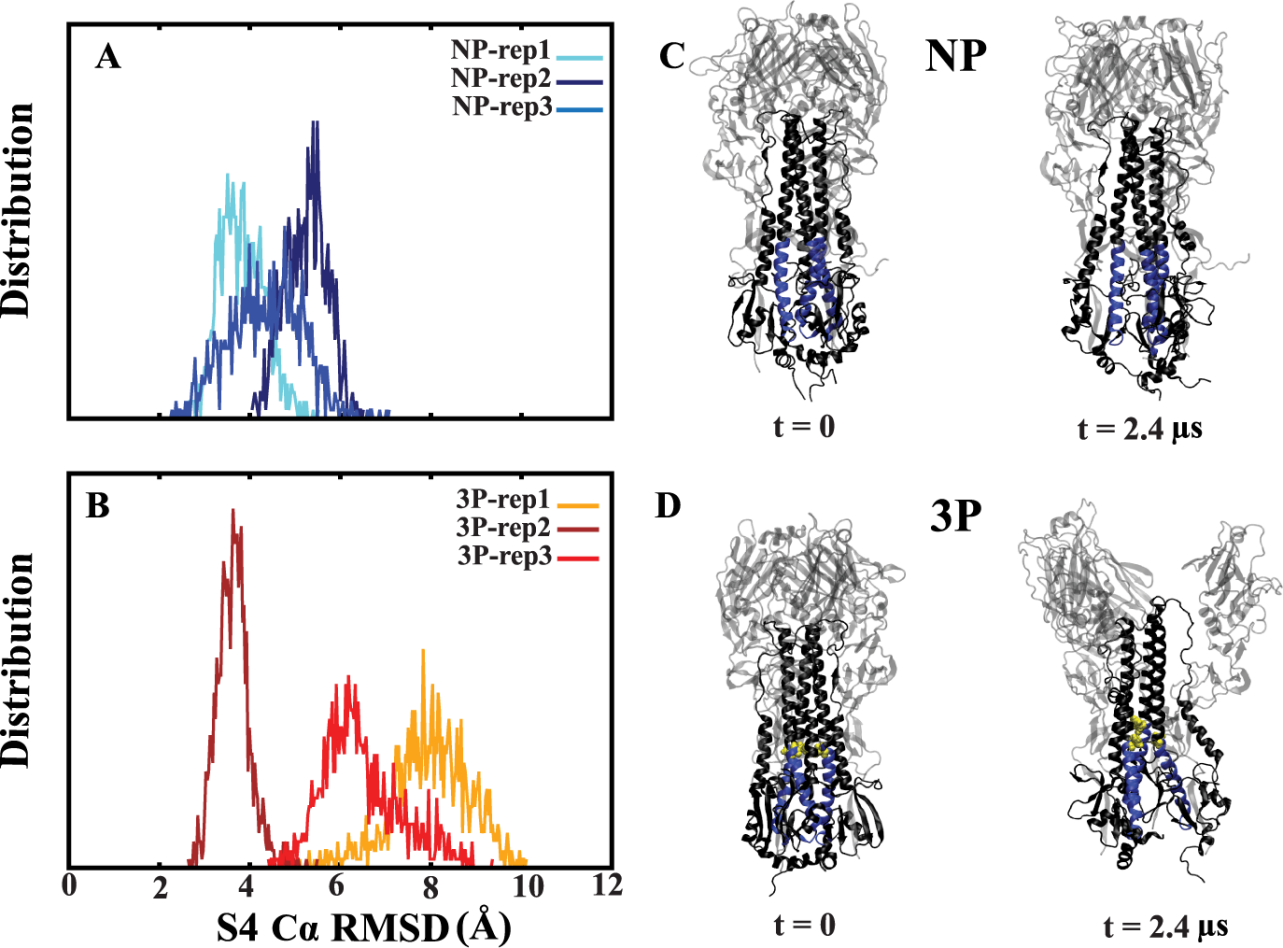
S4 Cα RMSD distributions. Probability distribution of S4 helices overall Cα RMSD values in nonprotonated (NP) (A) and fully protonated (3P) (B) systems during the final half of simulations (from 1.2 to 2.4 μs). The overall RMSD refers to aligning the entire protein and calculating the RMSD for the S4 domain specifically, providing insight into the flexibility of the S4 helices relative to the whole protein structure. (C, D) Cartoon representations of the NP and 3P system from the first and last frames of the simulation. The protonated histidine residues (H106_2_) are shown with the yellow color, while the S4 helices are represented in blue.

To determine the effect of protonated histidine on the deviation of the S4 helix, which includes a conserved histidine, we measured its Cα RMSD values (Figure S2). In the NP systems, the RMSD remained below 6 Å in all three repeats (Figure S2A). In contrast, in the 3P systems, the RMSD exceeded 6 Å in two out of three repeats (Figure S2B). We compared the last 1.2 μs of all repeats using RMSD distribution plots. The results indicate that the 3P systems exhibited greater deviation from the initial state, with RMSD values reaching up to 10 Å compared to 6 Å in the NP systems. This suggests that the protonation of histidine significantly impacts the conformational changes of the S4 helix (Figure 3).

**Figure 4:**
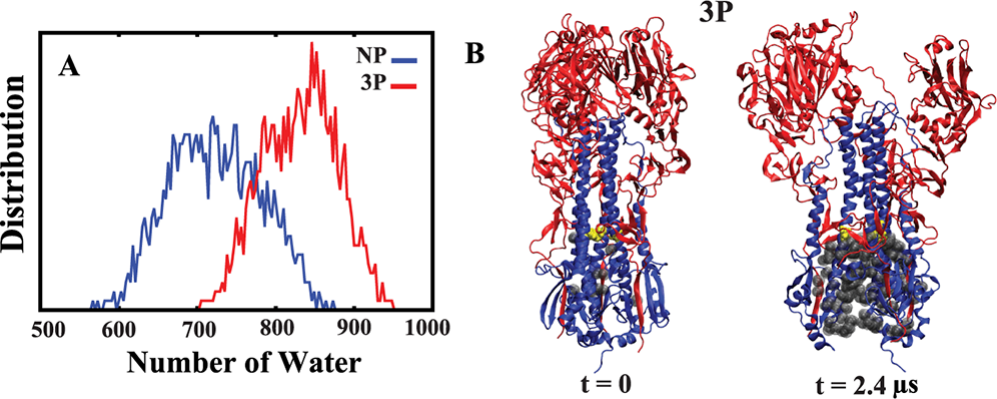
Water count probability distribution. (A) Probability distribution of the number of water molecules among three S4 helices during the final half of simulations (from 1.2 to 2.4 μs), comparing nonprotonated (NP) and fully protonated (3P) systems, based on three independent sets of simulations per condition. (B) Cartoon representation of the 3P system, showing the waters within the three S4 helices in the first and last frames of the simulation. Protonated histidines (H106_2_) and water molecules are shown in yellow and gray, respectively.

We also measured the number of water molecules in the lower region of the protein surrounding the three S4 domains (Figure S2C,D). The results show a higher concentration of water molecules in the fully protonated system (3P), especially after 1.2 μs of simulation. Distribution plots of water molecule counts during the last 1.2 μs indicate a peak of approximately 850 molecules in the 3P system, compared to around 700 molecules in NP system (Figure 4).

**Figure 5:**
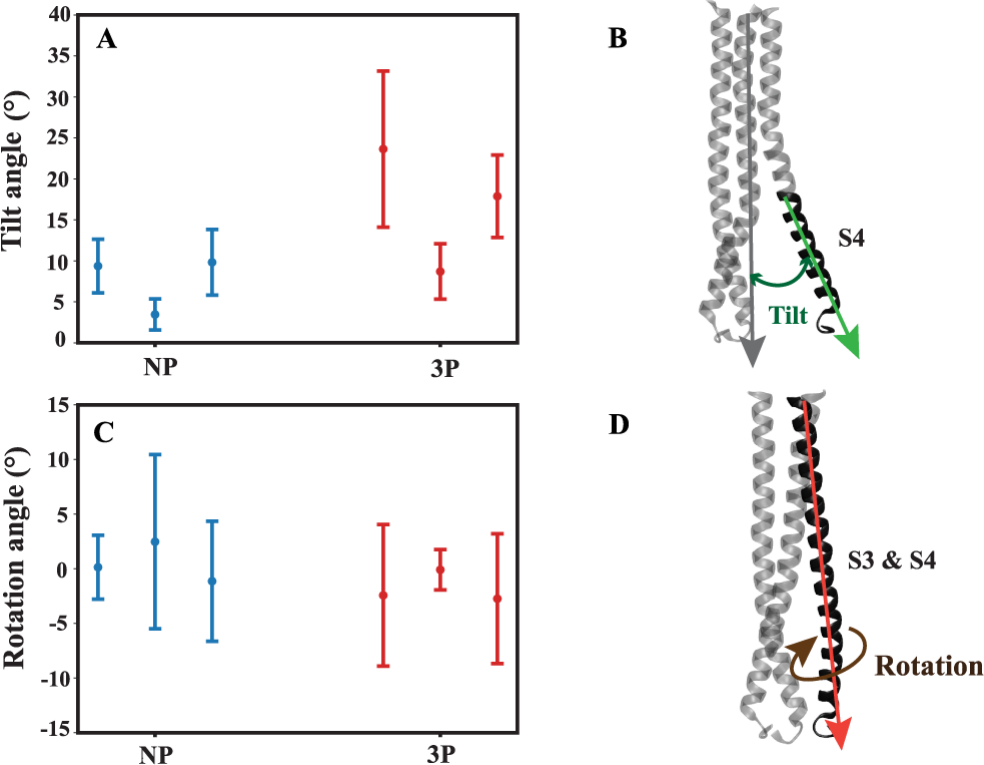
Average tilt and rotation angles. (A) The average tilt angle of the S4 helices for the last 1.2 μs of simulations in both nonprotonated (NP, blue) and fully protonated systems (3P, red), each repeated three times. (B) Definition of S4 tilt angle that is measured with respect to the entire long helices of HA2. (C) The average rotation angle of the long helices, including S3 and S4, for the last 1.2 μs in both nonprotonated (NP, blue) and fully protonated systems (3P, red), each repeated three times. (D) Definition of long helix rotation. The rotation of each helix is calculated with respect to its crystal structure.

Focusing on the tilt angle of S4 relative to the initial frame of HA2, we observed a contrasting trend between the 3P and NP systems. In all repeats of the 3P systems, the tilt angle consistently increased, while in the NP systems, it consistently decreased (Figure S3). This indicates a dynamic shift, where the S4 helices in the NP systems move closer to each other, while in the 3P systems, they move away from HA2. Furthermore, the average tilt angle analysis showed that the tilt angle of all three S4 domains was higher in the 3P system across the three repeats compared to the overall average in the NP system. A more dispersed data distribution in the 3P system also suggests a greater degree of opening in the fully protonated environment (Figure 5A).

We observed distinct rotational differences in the side chain of the conserved histidine (H106_2_) between protonated and nonprotonated systems. To illustrate this, we analyzed the angle of the long helix across all systems, focusing on both the S3 and S4 domains. In the 3P systems, the rotation angle (Figure S4) gradually decreased during the simulation, indicating a counterclockwise rotation in most cases. In contrast, the rotation angle either increased or remained stable in most NP systems, suggesting a clockwise rotation and an inward-facing conformation (Figure S4). Additionally, the plot in (Figure 5B) shows that the average rotation angle in the 3P systems, considering both S3 and S4 domains, was reduced compared to the NP systems. Our analysis of the tilt angle and rotational behavior clearly demonstrates that protonation plays a key role in shaping the conformational behavior of the S4 helix and the rotational dynamics of the conserved histidine (H106_2_).

In addition to our findings, relevant literature^83^ further supports the significance of our observations. A recent study on influenza hemagglutinin (HA) highlighted the elusive nature of intermediate structures in HA conformational dynamics. Using cryo-electron microscopy (cryo-EM), the study identified two distinct low pH intermediate conformations, revealing notable conformational changes in the central helices (S3 and S4). Specifically, these helices exhibited a counterclockwise rotation of 9.5° and a shift of 15 Å. The most significant changes were observed in the helices corresponding to helix Ds, aligning with our identification of the S4 region. This convergence of findings reinforces the relevance of our study and emphasizes the importance of understanding the dynamic behavior of HA in response to protonation.^83^

### Investigating Protonation Effects on Helix Rotation in Partially and Fully Protonated Systems

**Figure 6:**
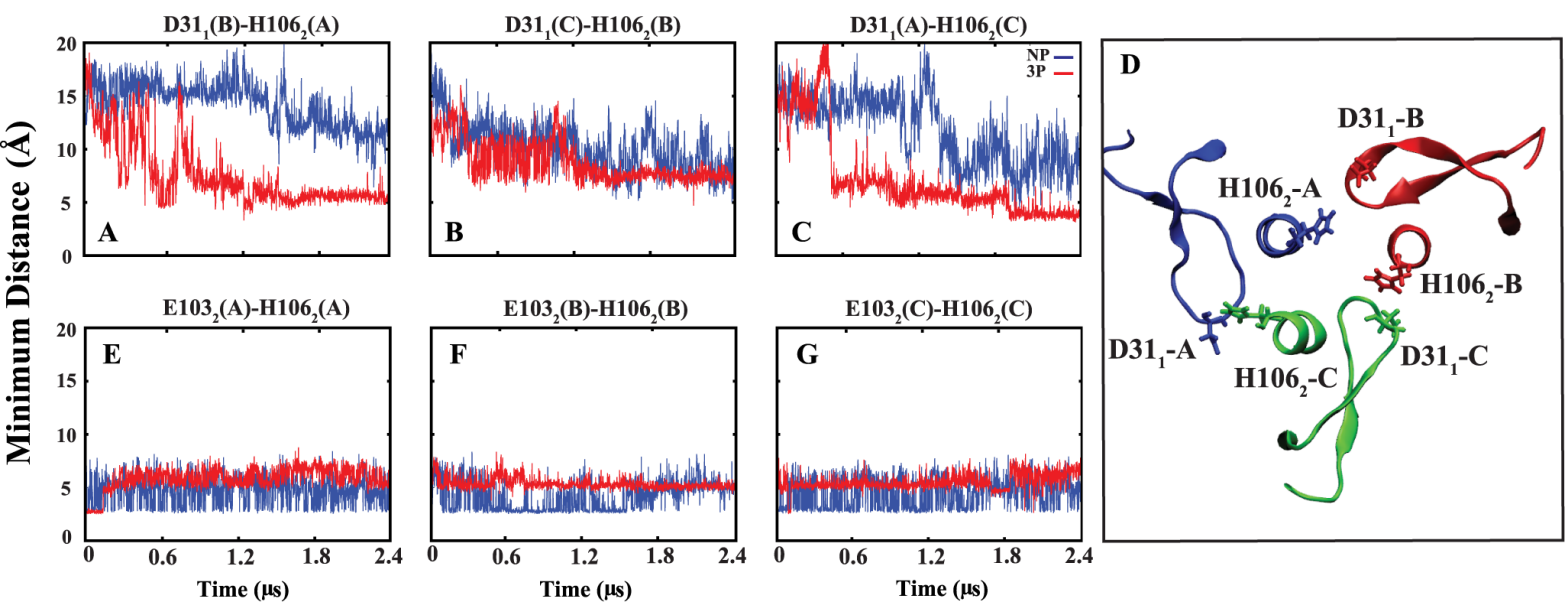
(A–C) Minimum distance between H106_2_ (HA2) and its neighboring D31_1_ (HA1). (D) Graphical representation of H106_2_ and D31_1_ in different monomer. (E–G) Minimum distance between H106_2_ and E103_2_, both located on the same monomer as HA2.

To further investigate the underlying reasons for the observed rotation, we conducted additional analyses, including salt bridge and hydrogen bond. The salt bridge analysis revealed a significant interaction between H106_2_ from HA2 and its neighboring HA1 residue (D31_1_), located in the 30-loop region. This bond appears to be absent in the nonprotonated (NP) systems (Figure 6A–D). This finding provides insights into the potential role of protonation in stabilizing key intermolecular interactions, thereby influencing the observed rotational dynamics within the system.

Measuring the minimum distance between D31_1_ and H106_2_ in all systems revealed interesting findings (Figure S5). In the NP systems, this distance consistently remains greater than 5 Å, indicating that a salt bridge is not formed. However, in the 3P systems, this distance was observed to decrease significantly in almost all simulation repeats, suggesting that a salt bridge could form under certain conditions. Although the distances in the current simulations are still somewhat larger than typical salt bridge distances, it is likely that with further simulation time, these distances could decrease further, potentially allowing for salt bridge formation. Additionally, analysis of the partially protonated systems (1P, 2P) displayed intermediate behavior, supporting the role of protonation states in modulating salt bridge formation and subsequent conformational dynamics (Figure S5).

An investigation by Benton et al. (2020)^27^ employed cryo-electron microscopy (cryo-EM) to examine structural transitions of the HA ectodomain at neutral and fusion pH. Their study highlighted the concerted rearrangements of HA1 and HA2, particularly focusing on the 30-loop (HA1 residues 22–37). They found that some interactions between the 30-loop and the long helix of HA2 were similar in states I (neutral) and IV (extended HA2). In state IV, side chain changes were observed, associated with the relocation of the short helix of HA2. Notably, in this state, the 30-loop formed interactions with H106_2_ and Q105_2_ at the site of the 180° turn in the fusion-pH structure, facilitating potential interactions between T30_1_ and Q105_2_, as well as H106_2_ of the adjacent HA2 chain. Additionally, the strictly conserved N104_2_ formed hydrogen bonds with loop residues K27_1_ and K315_1_ of HA1, as well as HA2 residue Q105_2_, suggesting important roles for the 30-loop in the refolding process. In our research, we observed similar trends in hydrogen bond occupancy between N104_2_ and K27_1_ and K315_1_ across different protonation states, with occupancy around 70% for both protonated and nonprotonated systems. However, our findings also highlight distinct interactions involving H106_2_, particularly with the neighboring HA1 residue D31_1_ (30-loop) in our protonated systems (Figure 6A–C).

Furthermore, in 3P systems, we observed that H106_2_ loses its interaction with E103_2_ of the same monomer HA2, whereas this interaction is more prominent in NP systems (Figure 6E–G). The minimum distance calculations between H106_2_ and E103_2_ show that this interaction was evident in the NP systems and in some cases of partially protonated systems (1P and 2P). However, in the 3P systems, this interaction either failed to form or was disrupted at the onset of the simulations (Figure S6). Interestingly, this salt bridge predominantly forms during equilibration states, especially in monomers lacking protonation. These observations provide additional insights into the conformational dynamics of HA under different pH conditions.

In a study by Milder et al. (2022),^84^ the authors investigated the stabilization of prefusion hemagglutinins (HAs) and their conformational impacts through the mutation of residues in the pH-sensitive region of HA2. By determining the X-ray crystal structure of the stabilized apo ectodomain of group 2 H3-HK68, they found that the mutations caused three histidines (H106_2_) to flip toward the trimer’s 3-fold axis, forming hydrogen bonds with D109_2_. Additionally, I29_1_ from the 30-loop was observed to lock histidine (H106_2_) in an inward-facing conformation by shifting toward the 3-fold axis.

### The Enhanced Probability of Fusion Peptide Release in Protonated Systems

**Figure 7:**
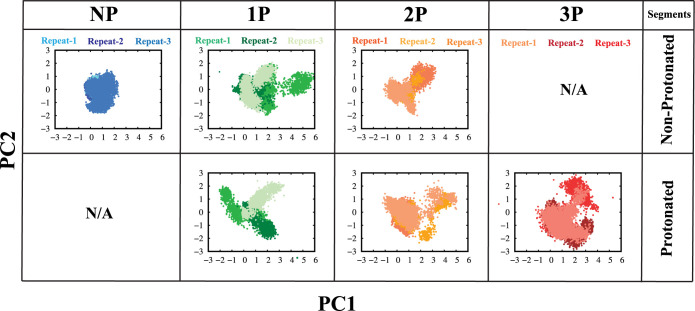
Projections of principal components (PCs) 1 and 2 depict the conformational landscape of the fusion peptide across nonprotonated (NP), partially protonated (1P, 2P), and fully protonated (3P) systems, as revealed by PCA. The first row represents nonprotonated segments in each system, while the second row displays protonated segments. Each color corresponds to a specific system. For comparison of the major modes of variation between protonated and nonprotonated conditions, PCA was performed separately for protonated and nonprotonated segments. This approach highlights the impact of histidine protonation on the conformational landscape of the fusion peptide.

An interesting aspect observed in our study revolves around the release of the FP within these systems. Despite the lack of direct observation of this event, strong evidence points to a significant difference in the stability of the FP between systems that are protonated and those that are not. Interestingly, indications point toward decreased stability of the FP in systems with protonation (fully or partially) compared to nonprotonated ones. This observation highlights the potential influence of protonation states on the dynamics and stability of critical viral fusion machinery, providing important insights into the interaction between molecular behavior and environmental conditions during viral entrance processes. The conformational dynamics of the FP across all systems were investigated utilizing principal component analysis (PCA), achieved through a combination of trajectories and projection onto the first and second principal components. As a result, systems with protonation exhibited distinct positions on the PCA plots, demonstrating divergence even within their nonprotonated segments (Figure 7). It is essential to acknowledge that the FP undergoes protonation changes during the fusion process, driven by acidification in late endosomes. These protonation changes could influence the FP’s observed conformational dynamics and stability. However, our current study did not account for these variations in protonation states.

**Figure 8:**
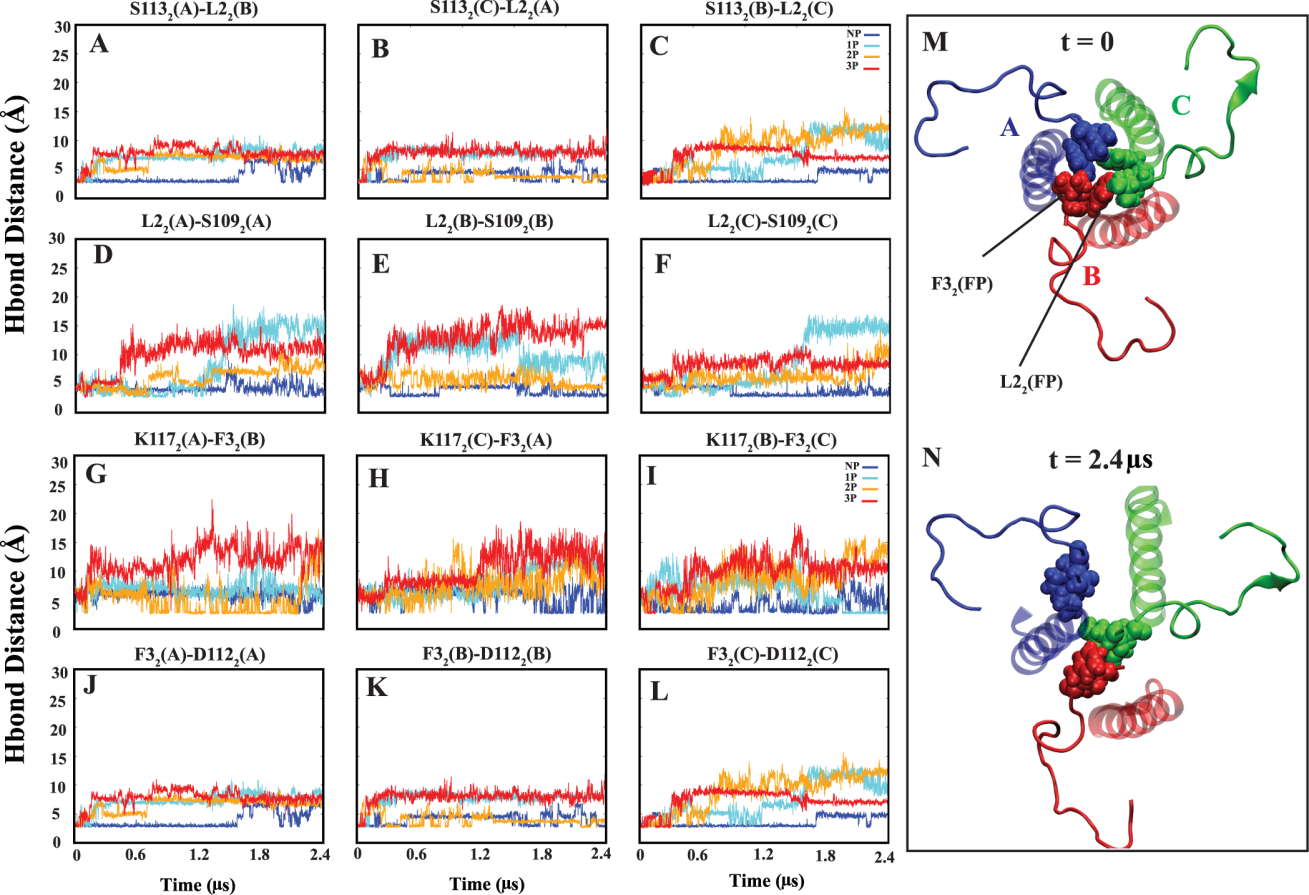
Inter- and intrahydrogen bond distances between FP residues (L2_2_, F3_2_) and S4 residues (S113_2_, D109_2_, K117_2_, and D112_2_). (A–C and G–I) Intermonomer hydrogen bonds between FP and S4 residues, indicating they are located in different monomers. (D–F and J–L) Intramonomer hydrogen bonds between FP and S4 residues, signifying they are located within the same monomer.

The hydrogen bond analysis revealed interactions involving the fusion peptide (FP) residue L2_2_ and neighboring residues, particularly S113_2_ and D109_2_ of S4 (Figure 8A–F). Interestingly, the interhydrogen bond between L2_2_ and S113_2_ (Figure 8A–C) showed higher occupancy in the nonprotonated (NP) system compared to the protonated ones, especially in the 3P system. Plotting these interactions demonstrated stability in NP systems, in contrast to their breakdown in partially or fully protonated systems (1P, 2P, and 3P). Additionally, an intrahydrogen bond interaction between L2_2_ and D109_2_ (Figure 8D–F) within the hinge region was observed, although it exhibited decreased occupancy in protonated systems. Consistent with earlier observations, the plots illustrated the disruption of this bond in nearly all protonated systems, while it remained stable in NP systems (Figures S7 and S8).

Another notable intrahydrogen bond interaction involves the fusion peptide (FP) residue F3_2_ and the neighboring S4 residue K117_2_ (Figure 8G–I). The average occupancy of this bond is nearly halved when comparing the NP and 3P systems. The hydrogen bond plots further support this observation, reflecting the trends noted in the average occupancy analysis. Similarly, an interhydrogen bond involving F3_2_ and another S4 residue, D112_2_ (Figure 8J–L), exhibited a decrease in average occupancy from NP to 3P systems. The hydrogen bond interaction plots for FP (F3_2_) and S4 (K117_2_, and D112_2_) are available in Figures S9 and S10.

In a recent investigation conducted by Gao et al (2020),^83^ the study focused on examining the structural intermediates during the low pH-induced transition of influenza hemagglutinin (HA). In the cryo-EM analysis of HA-antibody Fab complexes at neutral pH (pH 7.8), it was observed that the FP encircles the N-terminal fragment of HA1 (residues 9–19, HA1-N), forming a hydrogen bond with residue H17_1_ of HA1-N. Moreover, hydrophilic pockets are formed between the helix Ds (S4s), allowing the hydrophobic distal ends of the FPs to penetrate deeply, creating a hydrophobic core consisting of residues L2_2_ and F3_2_. Gao et al. employed cryo-EM and 3D classification techniques to characterize the structures of HA in low pH-induced intermediate states. Their findings indicate that exposure to low pH triggers significant conformational changes in HA, leading to the release of the fusion peptide.

### Partial Dissociation of HA1

**Figure 9:**
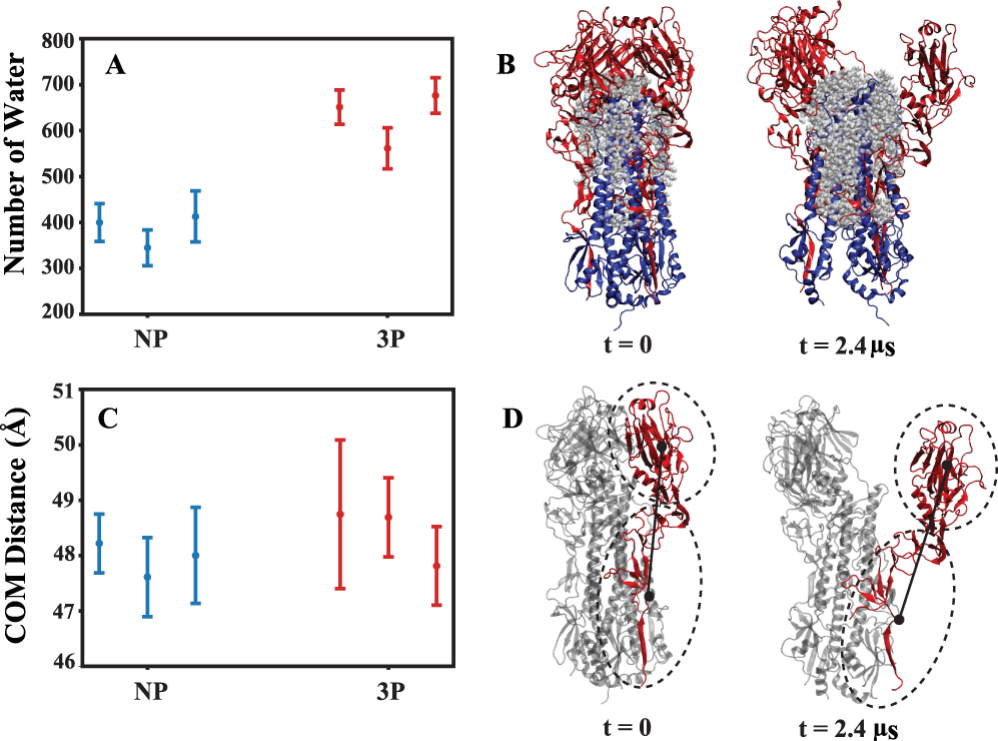
Average and standard deviation of penetrated water molecules and center of mass distances within each HA1 domain. (A) Number of water molecules between HA1 and HA2 in nonprotonated (NP) and fully protonated (3P) systems for each repeat, depicting the last 1.2 μs of the simulations. (B) Graphic representation of the first and last frames of 3P system, with water molecules surrounding HA1 (red) and HA2 (blue) depicted in gray. (C) Measurement of the distance between the center of mass of the head and tail of HA1 for each repeat, focusing on the last 1.2 μs of the simulations. (D) Molecular image showing the center of mass distance considered between the head and tail of each HA1.

Recent mesoscale simulations have highlighted the dynamic behavior of HA, specifically the ″breathing″ motion of the HA head. This breathing motion exposes previously hidden epitopes, suggesting increased plasticity and adaptability of the HA protein, which could be targeted by broadly neutralizing antibodies.^85^ Similar HA head breathing dynamics were also observed in earlier experimental studies.^86,87^ Additionally, several studies have demonstrated that low pH induces the initial conformational change in HA by inducing partial dissociation or detachment of HA1 subunits.^27,35,88−97^ In our study, we observed that protonation similarly affects HA1 detachment. It triggers the formation of a cavity between HA1 and HA2, allowing water to penetrate and interact with the HA2 domain that was previously protected. This interaction potentially enhances the flexibility of the HA2 domain. To demonstrate these changes, we conducted a comprehensive analysis of the number of water molecules between the HA1 and HA2 domains in both NP and 3P systems. Our findings reveal that the number of water molecules in the 3P systems is higher compared to the NP systems (Figure 9A,B), and the time dependent calculation of the water molecules is shown in Figure S11. We further investigated the movement of HA1 by calculating the center of mass distance between its head (RBD and VE) and tail (F′) (Figure 9C,D) to assess the impact of protonation. The (Supporting Information Figure S12) provides the time-dependent center of mass distance calculation. The center of mass distance between head and tail of the HA1 (Figure 9C) indicates that the 3P systems exhibit slightly greater movement compared to the NP systems. These results suggest that protonation potentially influences the movement of HA1 and affects the hydration dynamics between HA1 and HA2 domains.

Based on our hydrogen bond analysis, we identified three crucial hydrogen bonds that exhibit significant differences between the 3P and NP systems (Table 1). The table presents the actual occupancy values for these hydrogen bonds in each system. Here, we summarize the average occupancies for clarity. The first bond, between H17_1_ and H18_1_ located on HA1, shows an average occupancy of 60% in NP systems, decreasing to 40% in 3P systems. The second hydrogen bond formed between residues located on the 30-loop of HA1, G23_1_ and E35_1_, has an average interaction occupancy of 35% in NP systems, which decreases to 20% in 3P systems. The third hydrogen bond is between N322_1_ and V20_1_ of HA1 domain with an average occupancy of 59% in NP systems that reduces to 28% in 3P systems.

**Table 1:** Occupancy (%) of Inter-domain H-Bonds of HA1

	H17_1_–H18_1_ (%)			G23_1_–E35_1_ (%)			N322_1_–V20_1_ (%)		
System	rep. 1	rep. 2	rep. 3	rep. 1	rep. 2	rep. 3	rep. 1	rep. 2	rep. 3
**NP**	65	59	54	35	37	31	54	61	60
**3P**	33	44	41	18	25	17	19	35	30

A study^50^ highlights the importance of conserved histidine residues, particularly HA1 position 17 (H17_1_), in triggering acid-induced conformational changes in the influenza hemagglutinin (HA) protein. (H17_1_) is one of the conserved residues in the H3 subgroup, and extensive mutagenesis studies on these residues in H3 subtype HA suggest that (H17_1_) plays a significant role in inducing structural changes upon acidification. This indicates that (H17_1_) is involved in the pH-dependent conformational changes necessary for membrane fusion during viral entry.^50^

Both histidine and glutamate residues can accept protons on their side chains, leading us to hypothesize that the protonation of H106_2_ could increase the number of water molecules around these residues. This may result in decreased interactions between the residues and a higher probability of their protonation. In turn, this could create a more acidic environment, induce conformational changes in the protein, and enhance the likelihood of membrane fusion.

**Figure 10:**
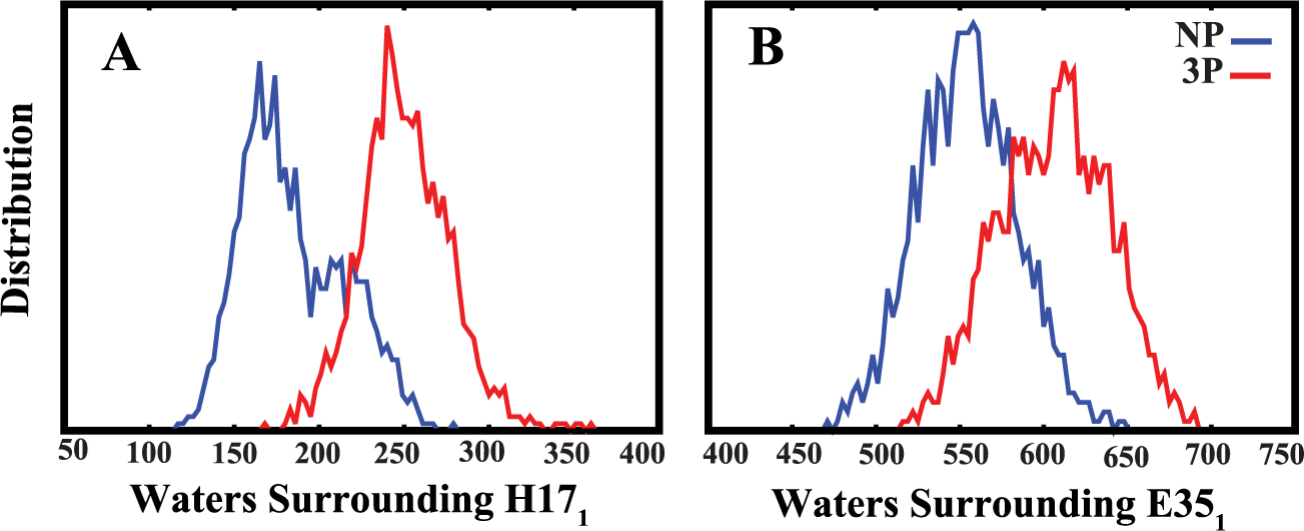
Comparing the number of water molecules surrounding the side chains of H17_1_ (A) and E35_1_ (B) in the HA1 domain, highlighting increased hydration in the fully protonated systems (3P). Data from all three repeats for each system are included in these plots, focusing on the last 1.2 μs of the simulations.

To assess the effect of surrounding water molecules on the interactions of these residues (H17_1_–H18_1_ and G23_1_–E35_1_), we analyzed the water molecules surrounding the side chains of H17_1_ and E35_1_. Our results revealed a significant increase in the number of water molecules around these residues during the last 1.2 μs of the simulation in the 3P systems compared to the NP systems (Figure 10A,B). The time series and correlation analysis of water around the side chains of these two residues are available in the (Supporting Information Figures S13 and S14).

## Conclusions

This work suggests that the protonation of H106_2_, a residue located in the hinge region of HA2, triggers conformational changes that could subsequently influence the fusion process of the HA protein. Our results indicate that protonating this specific residue induces notable conformational changes. These include decreased stability and the opening of the S4 helix, which contains the protonated histidine, along with an increase in water molecules between S4 helices. Additionally, H106_2_ residues exhibit an outward rotation in the protonated systems, whereas they maintain an inward-facing state in the NP systems. Furthermore, we observed initial signs of FP release and an increase in water molecules between HA1 and HA2. These changes trigger the conformational shifts in the protein that are necessary for facilitating the fusion process. Specifically, the conformational changes in HA1 include the center of mass distance between the head and tail of HA1 is altered in 3P systems compared to NP systems. The increased water penetration between HA1 and HA2 in the protonated systems disrupts hydrogen bonds, creating more space around capable residues to attract protons and creating a more acidic environment that enhances the probability of membrane fusion. Our findings highlight the critical role of H106_2_ protonation in HA protein-mediated fusion. However, further studies are needed to deepen our understanding of protonation’s role in the fusion process. In the context of molecular dynamics (MD) simulations, our research suggests that the protonation of H106_2_ can influence and potentially facilitate the fusion process.

## Supporting Information Available

Probability of p*K*_a_ exceeding 7.4 for conserved histidines (H106, H142, H159) (Figure S1); RMSD and water count analysis related to S4 (Figure S2); tilt angle of S4 helices (Figure S3); rotation angle of long helices, including S3 and S4 helices (Figure S4); minimum distance between H106_2_ and neighboring D31_1_ across protonation states (Figure S5); minimum distance between E103_2_ and H106_2_ in the same monomer of HA2 across protonation states (Figure S6); inter-hydrogen bond distance between FP (L2_2_) and S4 (S113_2_) in different monomers of HA2 across protonation states (Figure S7); intra-hydrogen bond distance between FP (L2_2_) and S4 (S109_2_) in the same monomers of HA2 across protonation states (Figure S8); inter-monomer hydrogen bond distance between FP (F3_2_) and S4 (K117_2_) in HA2 across protonation states (Figure S9); intra-monomer hydrogen bond distance between FP (F3_2_) and S4 (D112_2_) in HA2 across protonation states (Figure S10); water molecules between HA1 and HA2 (Figure S11); center of mass distance between head and tail of the HA1 domain (Figure S12); water molecules around side chains in various protonation states (Figure S13); correlation analysis of different protonation states (Figure S14) (PDF)
