# Supplementary material for: Molecular Dynamics Investigation of the Influenza Hemagglutinin Conformational Changes in Acidic pH
Source: J Phys Chem B. 2024 Nov 4;128(45):11151–63. doi: 10.1021/acs.jpcb.4c04607 (PMC11571222; doi:10.1021/acs.jpcb.4c04607)
Supplement: Supplementary file 1 — jp4c04607_si_001.pdf [file jp4c04607_si_001.pdf]

Supporting Information: Molecular Dynamics Investigation of the Influenza Hemagglutinin Conformational Changes in Acidic pH

Shadi A. Badiee, Vivek Govind Kumar, and Mahmoud Moradi*

Department of Chemistry and Biochemistry, University of Arkansas, Fayetteville, Arkansas 72701, U.S.A.

E-mail: moradi@uark.edu

List of Figures

S1 Probability of pKa exceeding 7.4 for conserved histidines (H106, H142, H159) S3

S2 RMSD and water count analysis related to S4. . . . . S4

S3 Tilt angle of S4 helices. S5

S4 Rotation angle of long helices, including S3 and S4 helices. S6

S5 Minimum distance between $H106_2$ and neighboring $D31_1$ across protonation states. . . . S7

S6 Minimum distance between $E103_2$ and $H106_2$ in the same monomer of HA2 across protonation states. S8

S7 Inter-hydrogen bond distance between FP ($L2_2$) and S4 ($S113_2$) in different monomers of HA2 across protonation states. S9

S8 Intra-hydrogen bond distance between FP ($L2_2$) and S4 ($S109_2$) in the same monomers of HA2 across protonation states. S10

S9 Inter-monomer hydrogen bond distance between FP ($F3_2$) and S4 ($K117_2$) in HA2 across protonation states. S11

S10 Intra-monomer hydrogen bond distance between FP ($F3_2$) and S4 ($D112_2$) in HA2 across protonation states. S12

S11 Water molecules between HA1 and HA2. . . . . S13

S12 Center of mass distance between head and tail of the HA1 domain. S14

S13 Water molecules around side chains in various protonation states. S15

S14 Correlation analysis of different protonation states. S16

	H106			H142			H159		
Systems	A	B	C	A	B	C	A	B	C
NP-rep1	0.98%	8.82%	39.22%	0.98%	0.00%	0.00%	0.00%	0.00%	0.00%
NP-rep2	0.00%	0.00%	6.86%	2.94%	0.98%	3.92%	0.00%	0.98%	4.90%
NP-rep3	4.90%	2.94%	4.90%	2.94%	1.96%	0.00%	0.00%	0.98%	0.00%

Fig. S1. Probability of pKa exceeding 7.4 for conserved histidines (H106, H142, H159). Probability of pKa values exceeding 7.4 for the conserved histidines (H106, H142, H159) across three protomers and three independent non-protonated simulation repeats (NP). H106 shows a significantly higher probability of exceeding a pKa of 7.4, reaching nearly 40% in at least one protomer and one repeat, while the other histidines display probabilities of no more than 5%.

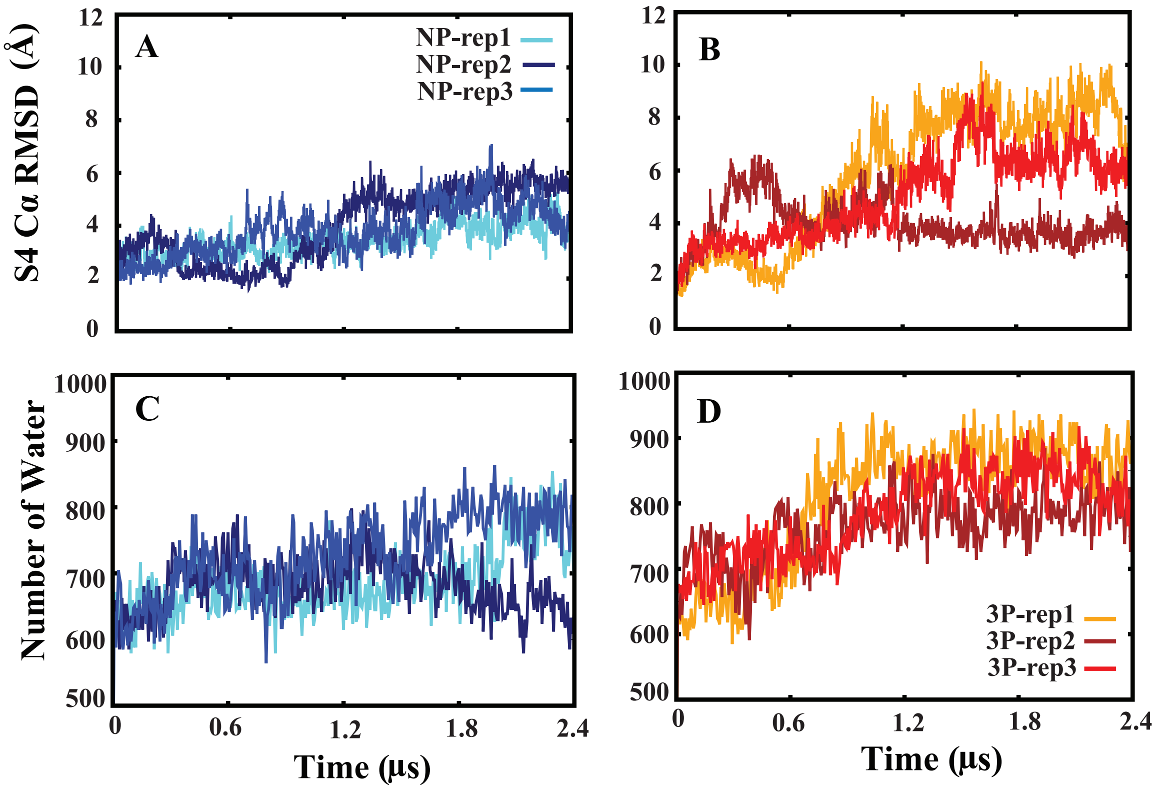

Fig. S2. RMSD and water count analysis related to S4. (A, B) The Cα RMSD calculated for the S4 domains relative to the initial structure over the 2.4 μs simulation is plotted for both the non-protonated (NP) and fully-protonated (3P) simulations. (C, D) The number of water molecules within 3 Å of the three S4 helices of HA2, with each S4 from one monomer, was calculated during the 2.4 μs simulation. Each repeat is indicated with a different color.

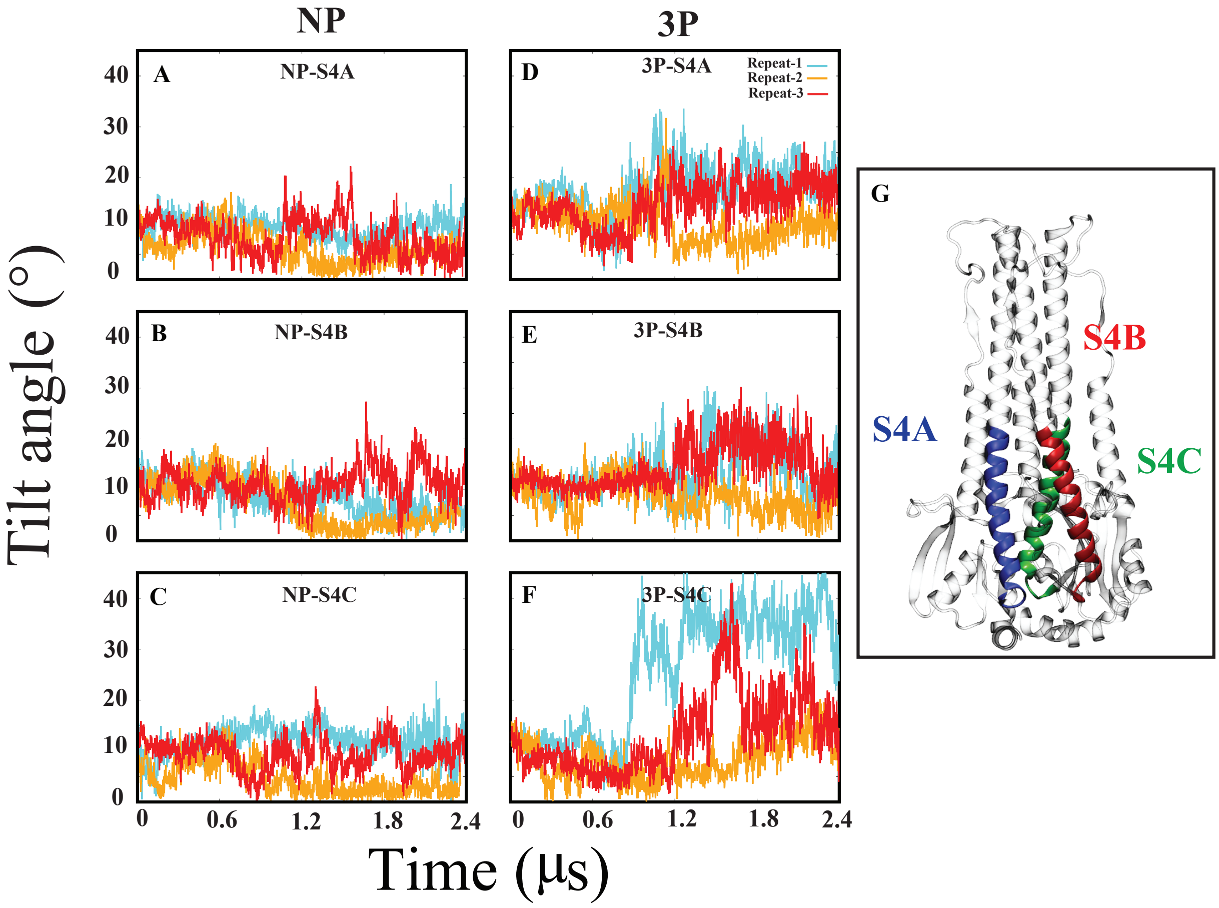

Fig. S3. Tilt angle of S4 helices. Time series of the tilt angle for S4 helix segments such as segment A (S4A), B (S4B), and C (S4C) in both non-protonated (NP) and fully-protonated (3P) systems. Each system undergoes three repetitions, with each simulation lasting 2.4 μs.

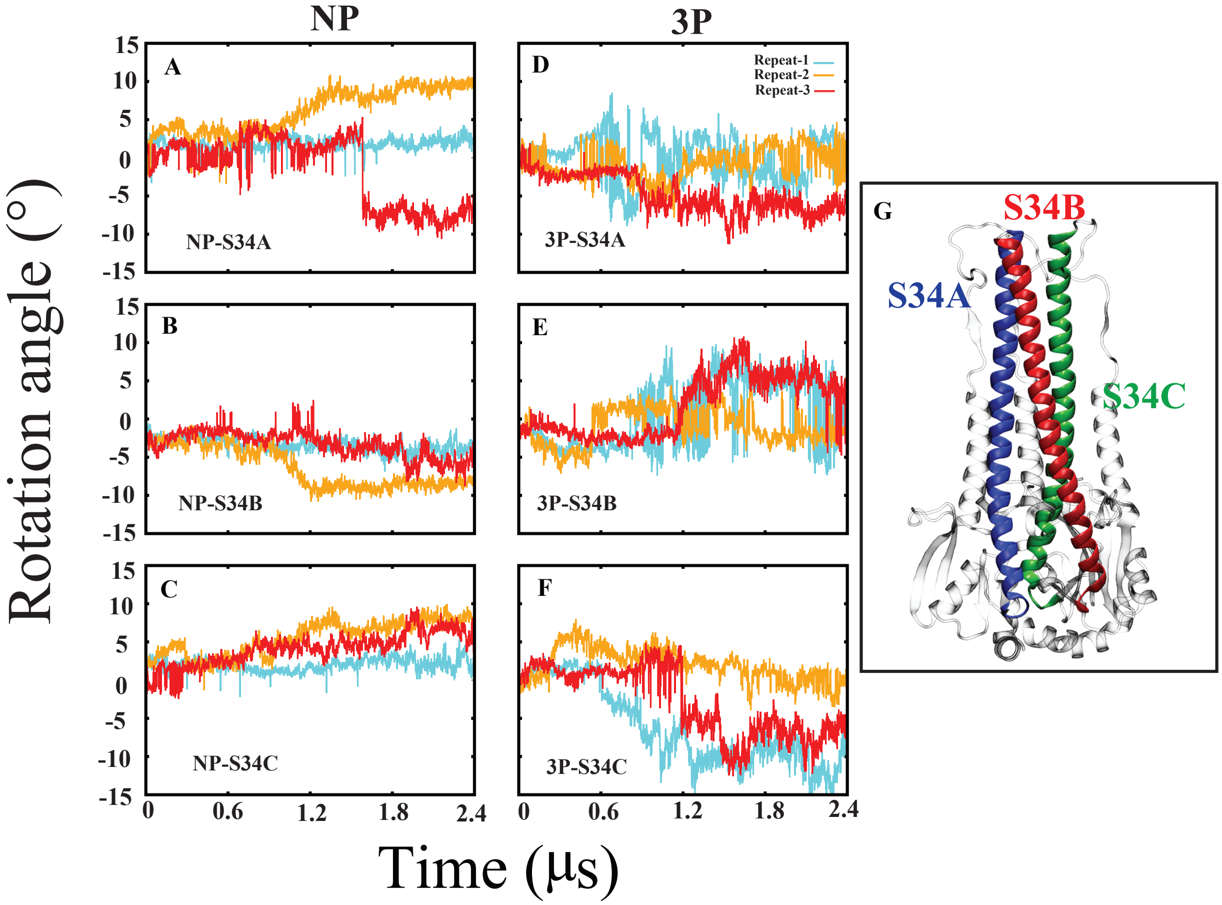

Fig. S4. Rotation angle of long helices, including S3 and S4 helices. Time series of the rotation angle for the long helix of HA2, including S3 and S4 helices, segmented into A (S34A), B (S34B), and C (S34C), in both non-protonated (NP) and fully-protonated (3P) systems. Each system is repeated three times during the 2.4 μs simulation.

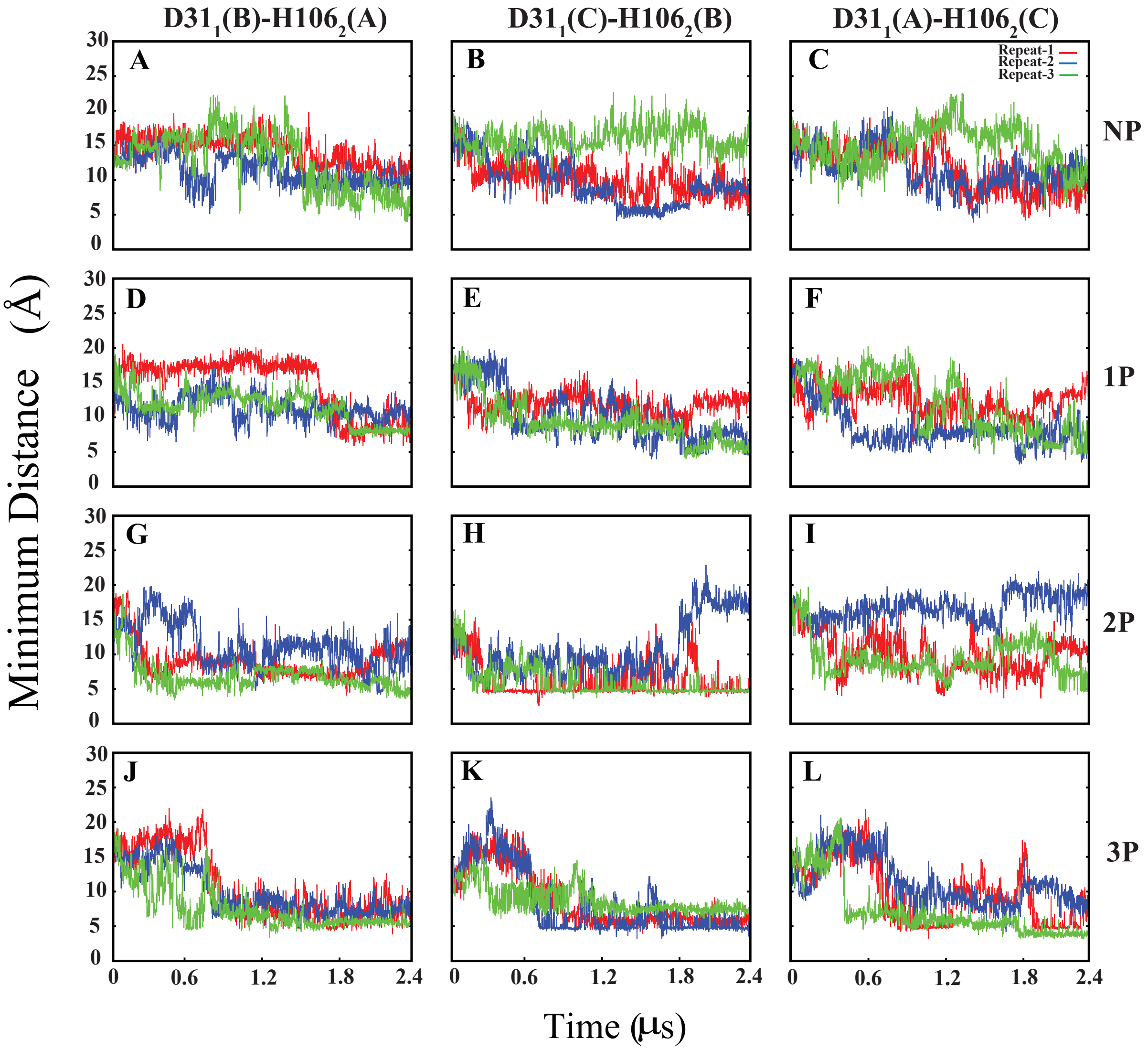

Fig. S5. Minimum distance between H106$_2$ and neighboring D31$_1$ across protonation states. Minimum distance between D31$_1$ and H106$_2$, located on HA1 and HA2, respectively. Rows represent different protonation states: non-protonated (NP), partially protonated (1P, 2P), and fully-protonated (3P). Each repeat is distinguished by a different color.

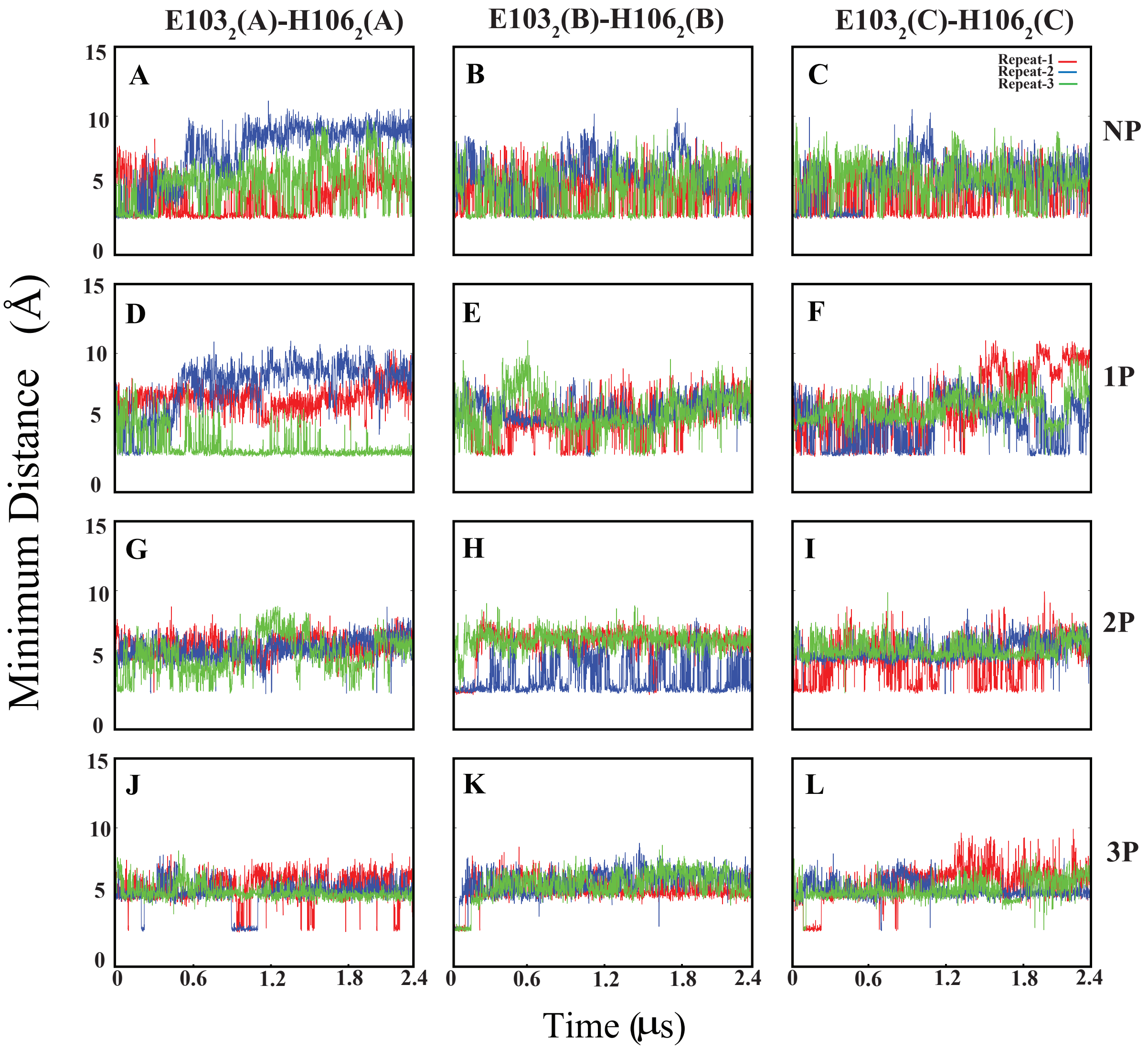

Fig. S6. Minimum distance between E103$_2$ and H106$_2$ in the same monomer of HA2 across protonation states. Minimum distance between E103$_2$ and H106$_2$, located on the same monomer of HA2. Rows represent different protonation states: non-protonated (NP), partially protonated (1P, 2P), and fully-protonated (3P). Each repeat is distinguished by a different color.

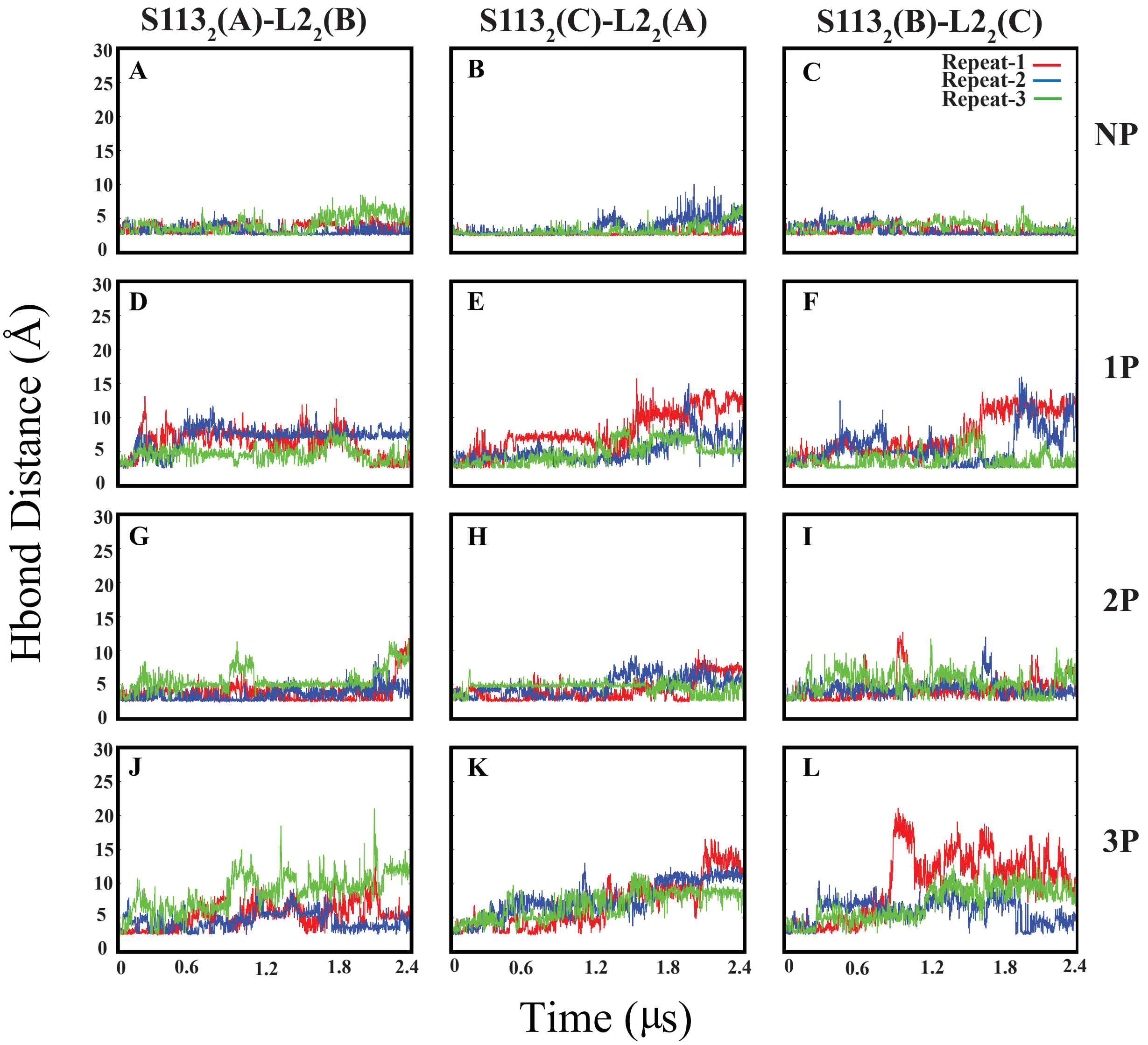

Fig. S7. Inter-hydrogen bond distance between FP ($L2_2$) and S4 ($S113_2$) in different monomers of HA2 across protonation states. Inter-hydrogen bond distance between $L2_2$ (FP) and $S113_2$ (S4), located on different monomers of HA2, during a 2.4 μs simulation. Rows represent different protonation states: non-protonated (NP), partially protonated (1P, 2P), and fully-protonated (3P). Each repeat is distinguished by a different color.

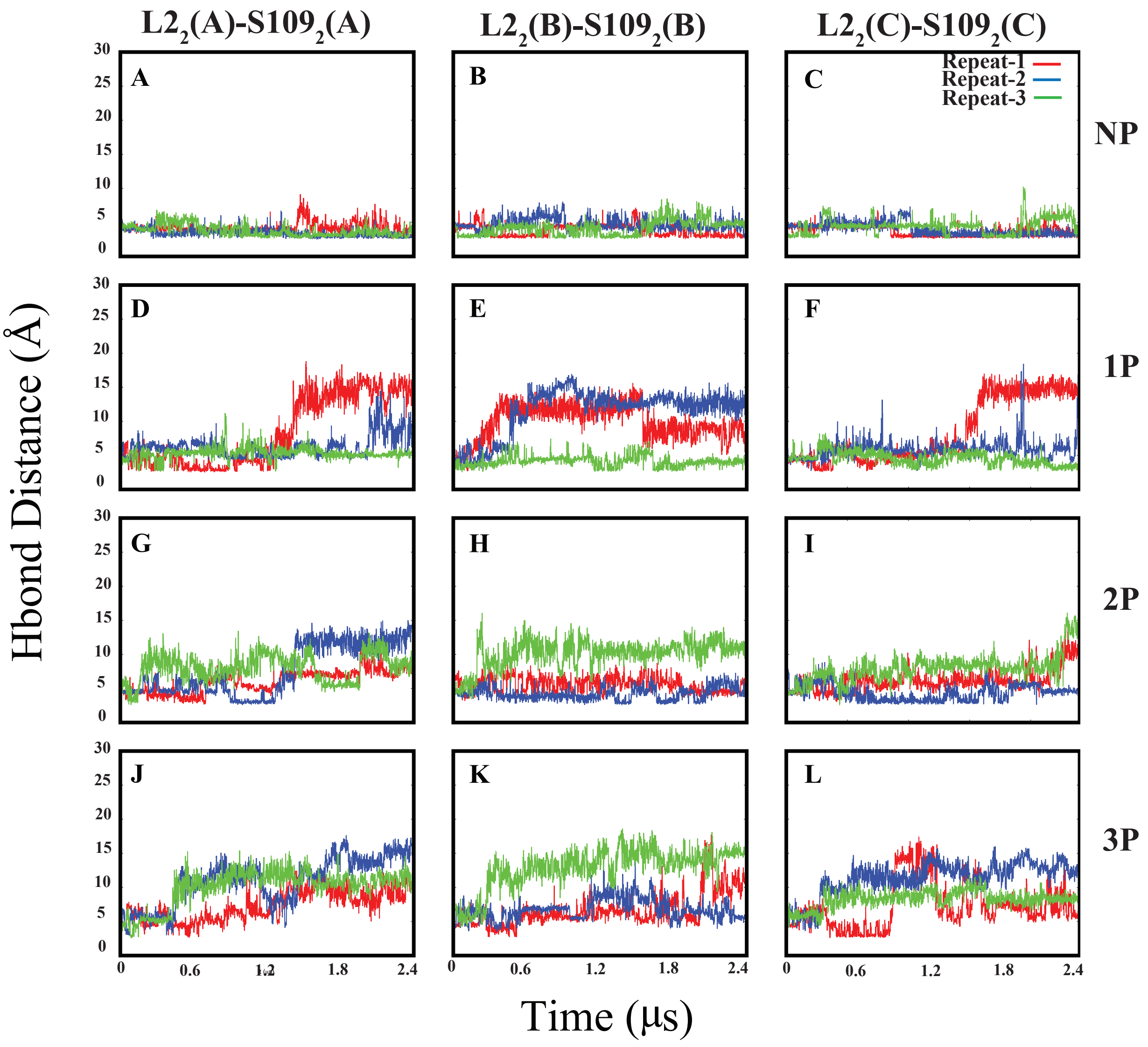

Fig. S8. Intra-hydrogen bond distance between FP ($L2_2$) and S4 ($S109_2$) in the same monomers of HA2 across protonation states. Intra-hydrogen bond distance between $L2_2$ (FP) and $S109_2$ (S4), located on the same monomers of HA2, during a 2.4 μs simulation. Rows represent different protonation states: non-protonated (NP), partially protonated (1P, 2P), and fully-protonated (3P). Each repeat is distinguished by a different color.

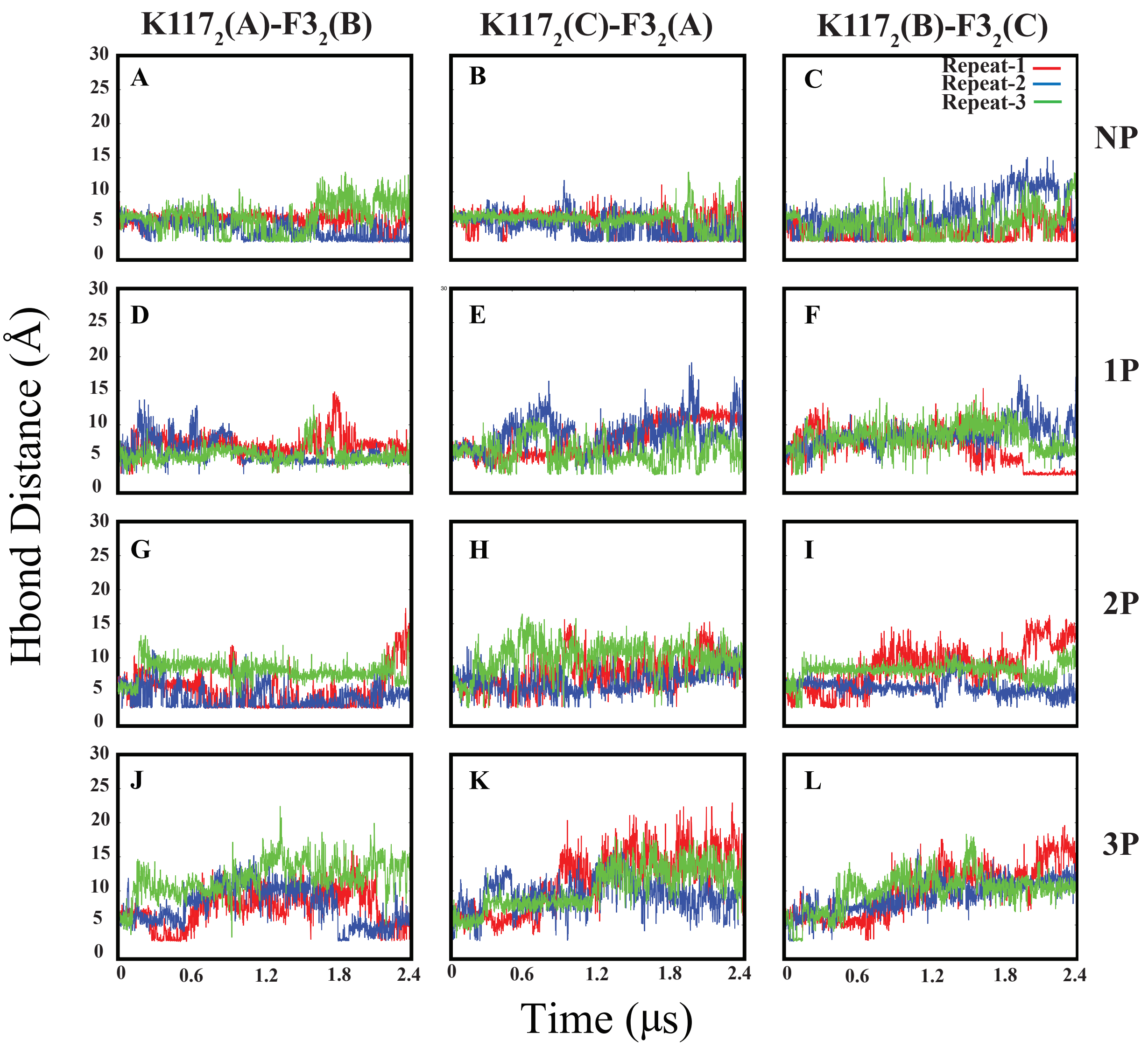

Fig. S9. Inter-monomer hydrogen bond distance between FP ($F3_2$) and S4 ($K117_2$) in HA2 across protonation states. Inter-monomer hydrogen bond distance between $F3_2$ (FP) and $K117_2$ (S4) in HA2, observed during a 2.4 μs simulation. Protonation states include non-protonated (NP), partially protonated (1P, 2P), and fully-protonated (3P), with each repeat distinguished by a different color.

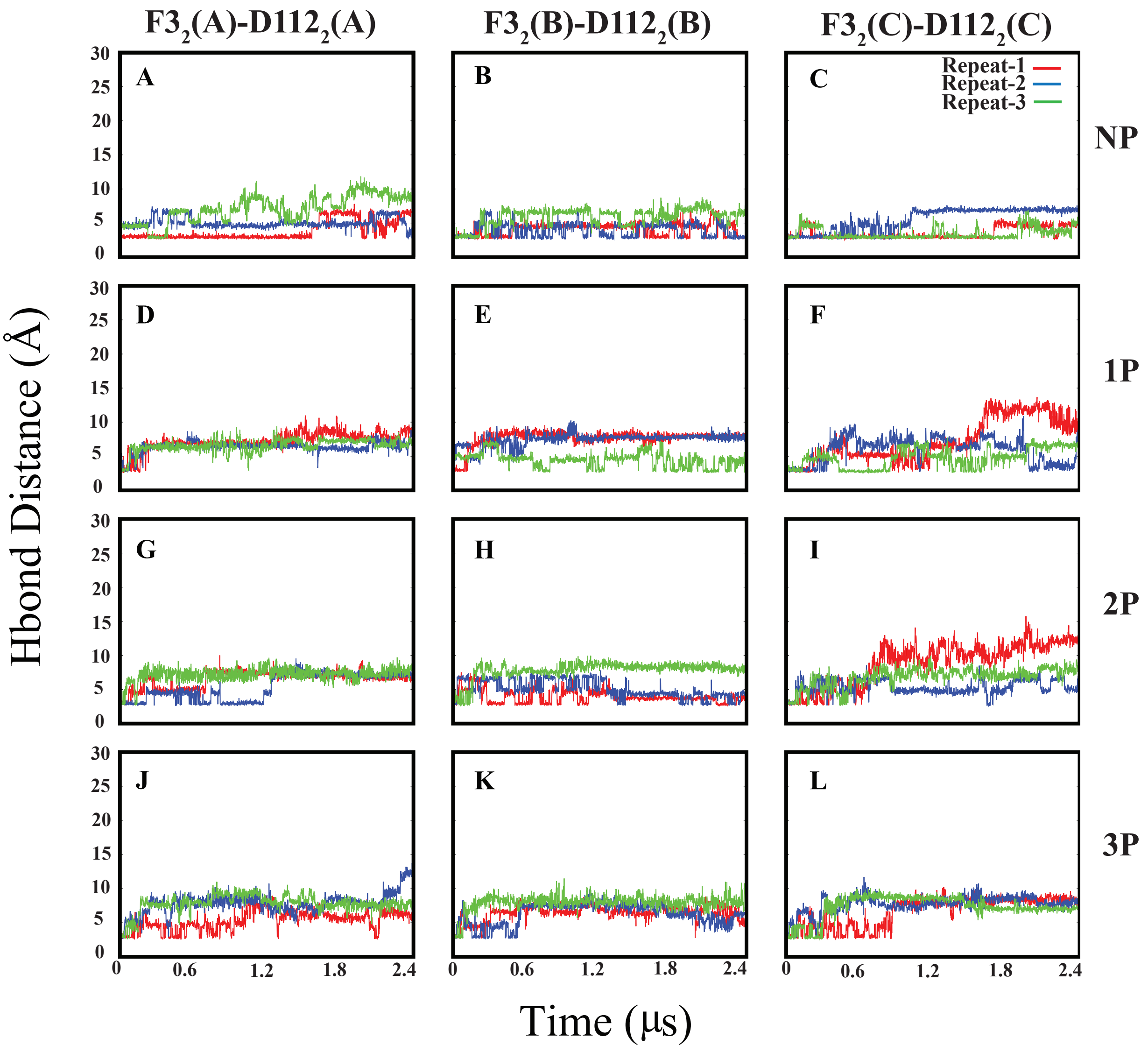

Fig. S10. Intra-monomer hydrogen bond distance between FP ($F3_2$) and S4 ($D112_2$) in HA2 across protonation states. Intra-hydrogen bond distance between $F3_2$ (FP) and $D112_2$ (S4), located on the same monomers of HA2, during a 2.4 μs simulation. Each row indicates different protonation states such as NP, 1P, 2P, and 3P, with each repeat distinguished by a different color.

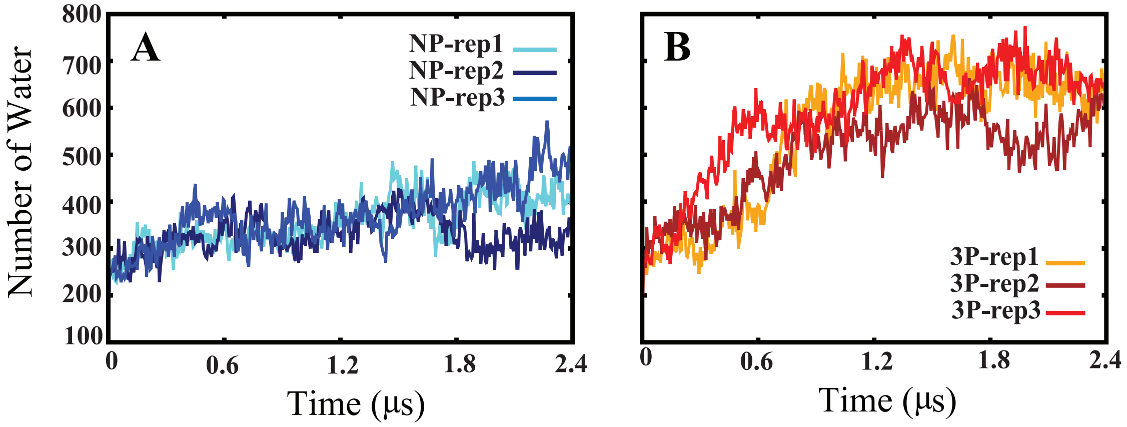

Fig. S11. Water molecules between HA1 and HA2. The number of water molecules calculated between HA1 and HA2 during the simulations in non-protonated (NP) and fully-protonated (3P) systems. Each repeat is indicated by a different color.

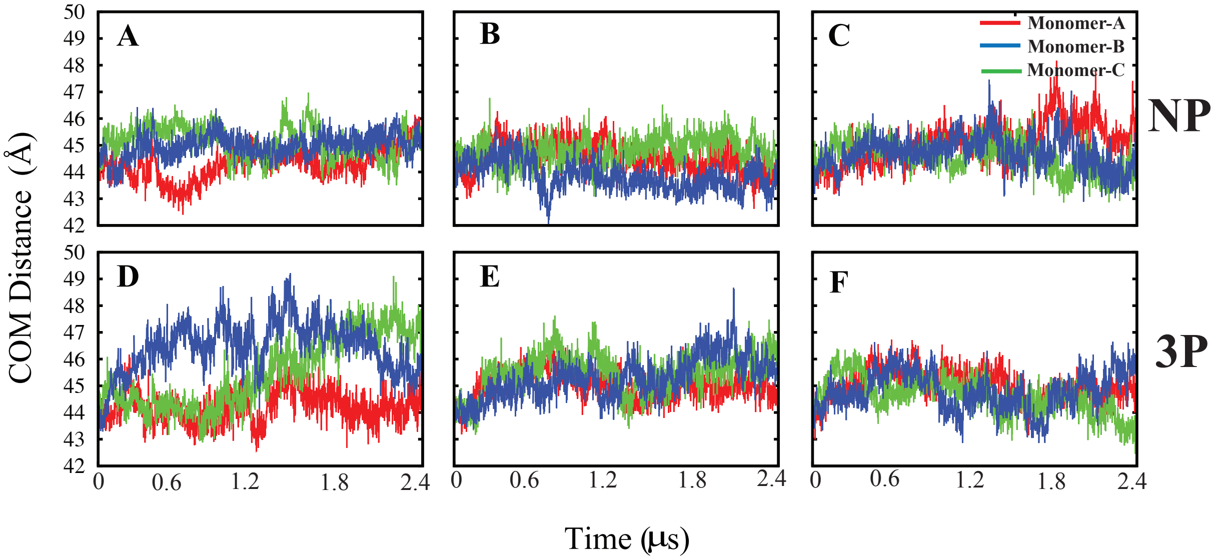

Fig. S12. Center of mass distance between head and tail of the HA1 domain. The center of mass distance between the head and tail of the HA1 domain during the simulations, with each monomer indicated by a different color.

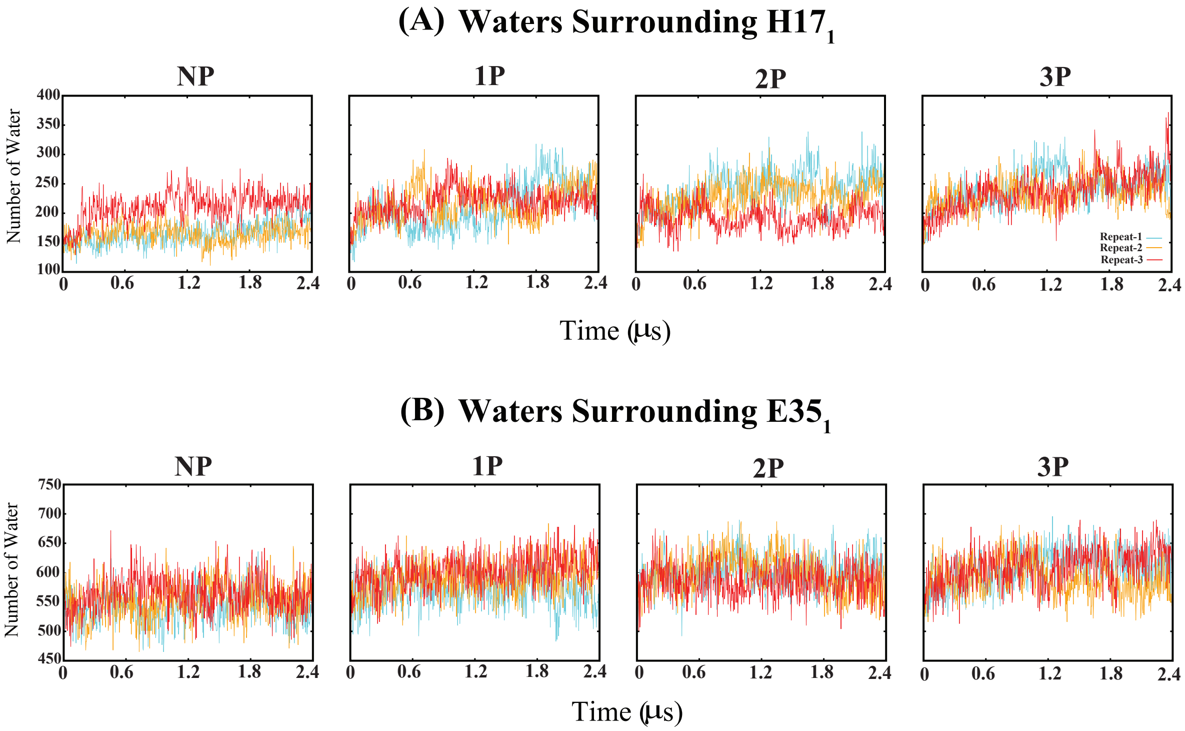

Fig. S13. Water molecules around side chains in various protonation states. The number of waters around the side chains of (A) residue H17$_1$ and (B) E35$_1$ calculated in non-protonated (NP), partially protonated (1P, 2P), and fully-protonated (3P) systems.

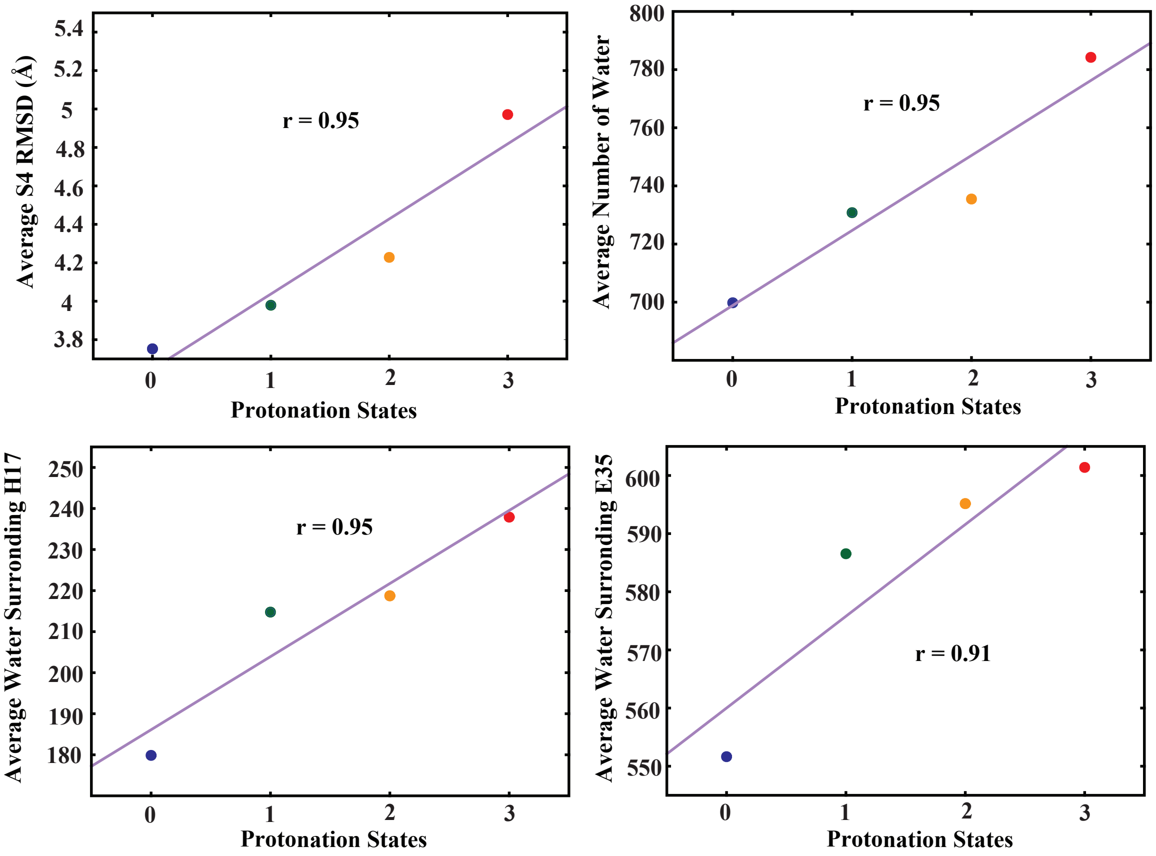

Fig. S14. **Correlation analysis of different protonation states.** Correlation analysis of different protonation states. This figure illustrates the average RMSD of S4, the average number of water molecules within S4, and the average number of water molecules surrounding residues H17 and E35 across all four protonation states (0, 1, 2, and 3). The results indicate a positive correlation between the protonation of H106 and the conformational dynamics of the protein, as evidenced by the increase in average values for these parameters. The correlation coefficient is presented as a numerical value, and a line has been fitted to the data points to visually represent the linear relationship between the protonation state and these changes.
